# The global, regional, and national economic consequences of stroke

**DOI:** 10.1161/STROKEAHA.123.043131

**Published:** 2023-07-27

**Authors:** Jakob V. E. Gerstl, Sarah E. Blitz, Qing Rui Qu, Alexander G. Yearley, Philipp Lassarén, Rebecca Lindberg, Saksham Gupta, Ari D. Kappel, Juan C. Vicenty-Padilla, Edoardo Gaude, Kunakorn C. Atchaneeyasakul, Shashvat M. Desai, Dileep R. Yavagal, Luca Peruzzotti-Jametti, Nirav J. Patel, Mohammed A. Aziz-Sultan, Rose Du, Timothy R. Smith, Joshua D. Bernstock

**Affiliations:** 1 Department of Neurosurgery, Brigham and Women’s Hospital, Harvard Medical School, Boston, MA; 2 Harvard Medical School, Boston, MA; 3 David H. Koch Institute for Integrative Cancer Research, Massachusetts Institute of Technology, Cambridge, MA; 4 Department of Clinical Neuroscience, Karolinska Institutet, Solna, Sweden; 5 Department of Neurosurgery, Boston Children’s Hospital, Harvard Medical School, Boston, MA; 6 Department of Neurosurgery, San Jorge Children & Women’s Hospital, Santurce, PR; 7 Pockit Diagnostics Ltd., Cambridge, UK; 8 Mission Thrombectomy - Global Access for Stroke Treatment, USA; 9 HonorHealth Research and Innovation Institute, AZ; 10 Department of Neurology, University of Miami & Jackson Memorial Hospitals, FL; 11 Department of Clinical Neurosciences and NIHR Biomedical Research Centre, University of Cambridge, Cambridge, UK; 12 Department of Metabolism, Digestion and Reproduction, Imperial College London

**Keywords:** stroke, macroeconomic burden, value of lost welfare (VLW), disability-adjusted life years (DALY)

## Abstract

**Background:**

An understanding of global, regional, and national macroeconomic losses caused by stroke is important for allocation of clinical and research resources. The authors investigated the macroeconomic consequences of stroke disease burden in the year 2019 in 173 countries.

**Methods:**

Disability-adjusted life year (DALY) data for overall stroke and its subtypes (ischemic stroke, intracerebral hemorrhage, and subarachnoid hemorrhage), were collected from the Global Burden of Disease (GBD) study 2019 database. Gross domestic product (GDP, adjusted for purchasing power parity [PPP]) data were collected from the World Bank; GDP and DALY data were combined to estimate macroeconomic losses using a value of lost welfare (VLW) approach. All results are presented in 2017 international US dollars adjusted for PPP.

**Results:**

Globally in 2019, VLW due to stroke was $2059.67 billion or 1.66% of the global GDP. Global VLW/GDP for stroke subtypes was 0.78% (VLW = $964.51 billion) for ischemic stroke, 0.71% (VLW = $882.81 billion) for intracerebral hemorrhage and 0.17% (VLW = $212.36 billion) for subarachnoid hemorrhage. The Central European, Eastern European, and Central Asian GBD super-region reported the highest VLW/GDP for stroke overall (3.01%), ischemic stroke (1.86%) and for subarachnoid hemorrhage (0.26%). The Southeast Asian, East Asian, and Oceanian GBD super-region reported the highest VLW/GDP for intracerebral hemorrhage (1.48%).

**Conclusions:**

The global macroeconomic consequences related to stroke are vast even when considering stroke subtypes. The present quantification may be leveraged to help justify increased spending of finite resources on stroke in an effort to improve outcomes for stroke patients globally.

## Graphical abstract

**Graphical Abstract:**
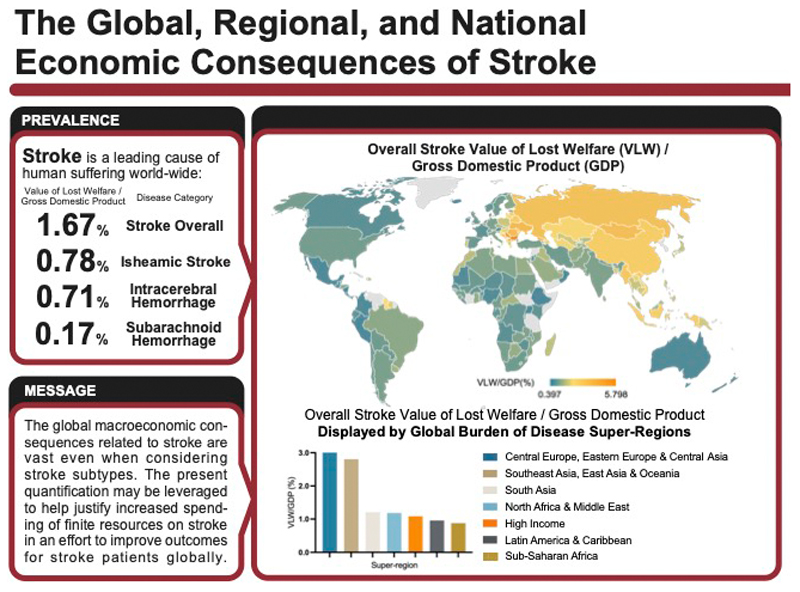

## Abbreviations and Acronyms

DALYs: Disability-adjusted life years
LMICs: Low- and middle-income countries
GBD: Global Burden of Disease
VLW: Value of lost welfare
VSL: Value of statistical life
WHO: World Health Organization
GDP: Gross domestic product
PPP: Purchasing Power Parity
USD: United States Dollar
IE: Income Elasticity
CHEERS: Consolidated Health Economic Evaluation Reporting Standards

## Introduction

In 2019, the global incidence of stroke was 12 million, prevalence 101 million, and mortality 7 million.^1^ The resulting disease burden of 143 million disability-adjusted life years (DALYs), makes stroke one of the leading causes of human suffering worldwide.^1,2^ The disease burden due to stroke also results in significant productivity losses.^3^ With populations ageing and many low- and middle-income countries (LMICs) transitioning from infectious to non-communicable disease(s) as their primary drivers of disease burden, the negative consequences of stroke are expected to increase even further.^4^ To counter such increases, effective allocation of limited resources is crucial; to do so requires a firm understanding of both epidemiological and macroeconomic trends associated with stroke.^5,6^

The Global Burden of Disease (GBD) study successfully maps epidemiological trends by providing readily available data on regional and country-wide stroke incidence, mortality, and disease burden in DALYs.^1,7^ In the 2019 study, GBD collaborators extended their analyses to assess disease burden for three subtypes of stroke (i.e., ischemic stroke, intracerebral hemorrhage, and subarachnoid hemorrhage).^1^ Despite this body of work, assessments related to the macroeconomic impact of stroke on regional and/or national economies remain sporadic.^8–10^ In fact, to the authors’ knowledge, no study has yet assessed the macroeconomic consequences of stroke and/or pertinent subtypes globally in a standardized manner. Given that the absence of such data is known to deter policy priority and attention,^11^ such an analyses may ultimately be leveraged to help drive resource allocation, and in so doing, improve stroke patient care globally.

The value of lost welfare (VLW) approach is an increasingly established model used to estimate economic losses caused by present disease burden in a standardized manner.^12–20^ The VLW model combines DALYs and the concept of value of statistical life (VSL), broadly defined as the value an individual is willing to pay to decrease the risk of mortality.^21^ Combined, these measures allow for the assessment of the macroeconomic consequences of a given disease cause.^21^ Moreover, the incorporation of VSL allows the VLW approach to assess economic welfare losses including non-market goods and services, and the value individuals attach to health itself (i.e. the value of being in a healthy state).^21^ Given the comprehensive nature of the resultant estimates, willingness-to-pay approaches such as the VLW have been promoted by the World Health Organization (WHO) when conducting macroeconomic modelling in health.^22,23^

Accordingly, herein, the authors estimate the macroeconomic losses attributable to stroke overall and its subtypes in 2019 in 173 high-, middle-, and low-income countries via GBD study DALY data.

## Methods

### Data sources

The data that support the findings of this study are available from the corresponding author upon reasonable request.

Using the GBD study database, DALY data related to stroke were obtained for 2019;^24^ as more recent DALY data are not yet available. Stroke overall, as defined by the GBD study includes ICD-10 codes I60.0-I60.9 (I60 = Subarachnoid hemorrhage), I61.0-I61.9 (I61=Intracerebral hemorrhage) I63.0-I63.9 (I63=Cerebral infarction), I64 (I64=Stroke, not specified as hemorrhage or infarction) and I69.0, I69.1, I69.3 (I69 = Sequelae of cerebrovascular disease) (Supplemental Table 1). Age-specific DALY data rates per year per 100,000 people were obtained for 173 countries.^25^ Gross domestic product (GDP) data and GDP per capita data, with purchasing power parity (PPP) adjustment to the 2017 United States Dollar (USD) for each country were gathered from the World Development Indicator Database, provided by the World Bank.^26^ All results are presented in 2017 international dollars adjusted for PPP.

GBD study super-regions were used to group countries as has been done previously.^25^ There are seven GBD study super-regions: 1) Central Europe, Eastern Europe, and Central Asia; 2) High-Income; 3) Latin America and Caribbean; 4) North Africa and Middle East; 5) South Asia; 6) Southeast Asia, East Asia, and Oceania; 7) sub-Saharan Africa.^25^

### Calculation of VLW

As defined briefly above, the VLW incorporates the concept of VSL which represents the maximum dollar value an individual is willing to pay to prevent mortality.^21^ Combined with DALYs of a particular disease, VSL can be used to estimate the total macroeconomic consequences of a given disease.^12,21^ VSL is determined empirically and has only been defined in a subset of mostly high income countries. To estimate VSL for all countries in a standardized manner, the following formula was employed using known estimates of VSL provided by the United States Department of Transportation:^12,20^

$$VSL_{peak,i}=VSL_{peak,USA}\frac{GDP_{i}{\;}^{IE}}{GDP_{USA}}$$

The GDP per capita of a specific country is in this way adjusted to that of the United States after PPP adjustment. Further adjustment for willingness to pay, can be done with a parameter known as income elasticity (IE) of the VSL.^27^ The gold standard IE when converting between high income regions has been 0.55; however, more conservative IEs of 1.0 and 1.5 have been used when converting from high income to low-income settings.^12,20,21^ An IE of the VSL of >1 assumes that individuals in high income settings are willing to pay disproportionately more to mitigate the risk of mortality than in lower income settings. To minimize assumptions about willingness to pay after adjustment for GDP per capita and purchasing power, an IE of 1.0 was selected for this study. Supplemental country-by-country analyses using IEs of 0.55 and 1.5 were, however, conducted to allow readers to use local willingness to pay assumptions after income adjustment. VSL_peak_ represents the age at which individuals in an economy are willing to pay the most to decrease the risk of mortality which has been empirically found to occur around middle age.^21^ To estimate VSL for any individual year (VSLY), VSL_peak_ was adjusted using a function known as f(a) which accounts for the estimated willingness to pay during different years of life, where *a* represents age, and f(a) is a quadratic function that adjusts a country’s peak VSL to VSL_a_ based on the proportion of life lived.^21^ Assuming imperfect capital markets, younger lower-income workers are not able to borrow against either idiosyncratic labor market shocks or borrow against higher future expected earnings. VSL is, therefore, depressed at younger ages. At older ages, the commodity bought through risk reduction is less than for young people, explaining the drop in value of VSL at older age. VSLYs were multiplied by age specific DALYs and subsequently summed to give the final VLW in USD (2017, PPP).^21^

All calculations were performed using RStudio IDE (RStudio, PBC, Boston, MA, USA). The work presented followed the Consolidated Health Economic Evaluation Reporting Standards (CHEERS) guidelines.^28^

### Ethics statement

Given that this study makes use of publicly available de-identified data aggregated at a country level, it is considered exempt as non-human subjects research by our Institutional Review Board.

## Results

**Figure 1:**
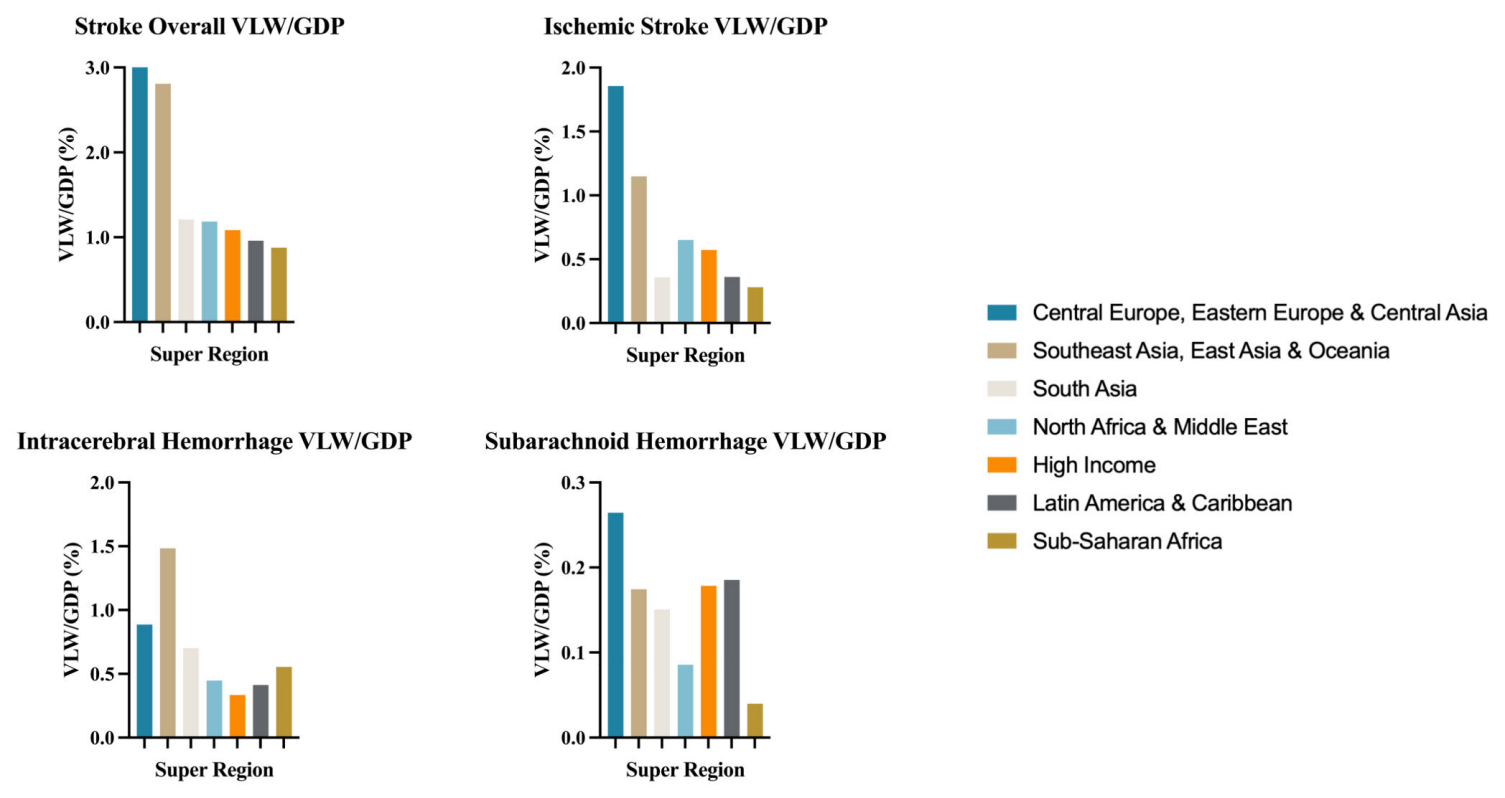
VLW/GDP in 2019 by GBD super-region for stroke overall, ischemic stroke, intracerebral hemorrhage, and subarachnoid hemorrhage. VLW = Value of Lost Welfare, GDP = Gross Domestic Product.

Globally in 2019, VLW due to stroke overall was $2059.67 billion or 1.66% of the global GDP. VLW as a share of GDP due to stroke overall was highest in the Central European, Eastern European, and Central Asian super-region (VLW/GDP = 3.01%; VLW = $285.82 billion) and in the Southeast Asian, East Asian and Oceanian super-region (VLW/GDP = 2.81%; VLW = $853.46 billion). Stroke overall had a VLW/GDP of 1.21% in the South Asian super-region (VLW = $133.82 billion); 1.18% in the North African and Middle Eastern super-region (VLW = $111.20 billion); 1.08% in the High-Income super-region (VLW = $565.14 billion); 0.96% in the Latin American and Caribbean super-region (VLW = $78.19 billion); and 0.88% in the sub-Saharan African super-region (VLW = $32.04 billion) (Figure 1).

Global VLW due to ischemic stroke in 2019 was $964.51 billion or 0.78% of the global GDP. VLW as a share of GDP due to ischemic stroke was the highest in the Central European, Eastern European, and Central Asian super-region (VLW/GDP = 1.86%; VLW = $176.47). Ischemic stroke had a VLW/GDP of 1.15% in the Southeast Asian, East Asian and Oceanian super-region (VLW = $291.91 billion); 0.65% in the North African and Middle Eastern super-region (VLW = $61.09 billion); 0.57% in the High-Income super-region (VLW = $298.39 billion); 0.36% in the Latin American and Caribbean super-region (VLW = $29.44 billion); 0.36 in the South Asian super-region (VLW = $39.54 billion); and 0.28% in the sub-Saharan African super-region (VLW = $10.29 billion) (Figure 1).

Global VLW due to intracerebral hemorrhage in 2019 was $882.81 billion or 0.71% of the global GDP. VLW as a share of GDP due to intracerebral hemorrhage was the highest in the Southeast Asian, East Asian and Oceanian super-region (VLW/GDP = 1.48%; VLW = $451.17 billion). Intracerebral hemorrhage had a VLW/GDP of 0.89% in the Central European, Eastern European, and Central Asian super-region (VLW = $84.21 billion); 0.70% in the South Asian super-region (VLW = $77.61 billion); 0.55% in the sub-Saharan African super-region (VLW = $20.29 billion); 0.45% in the North African and Middle Eastern super-region (VLW = $42.07 billion); 0.41% in the Latin American and Caribbean super-region (VLW = $33.62 billion); and 0.33% in the High-Income super-region (VLW = $173.84 billion) (Figure 1).

Global VLW due to subarachnoid hemorrhage in 2019 was $212.36 billion or 0.17% of the global GDP. VLW as a share of GDP for subarachnoid hemorrhage was highest in the Central European, Eastern European and Central Asian super-region (VLW/GDP = 0.26%; VLW = $25.15 billion). Subarachnoid hemorrhage had a VLW/GDP of 0.19% in the Latin American and Caribbean super-region (VLW = $15.12 billion); 0.18% in the High-Income super-region (VLW = $92.90 billion); 0.17% in the Southeast Asian, East Asian, and Oceanian super-region (VLW = $53.00 billion); 0.15% in the South Asian super-region (VLW = $16.68 billion); 0.09% in the North African and Middle Eastern super-region (VLW = $8.05 billion); and 0.04% in the sub-Saharan African super-region (VLW = $1.46 billion) (Figure 1).

**Figure 2:**
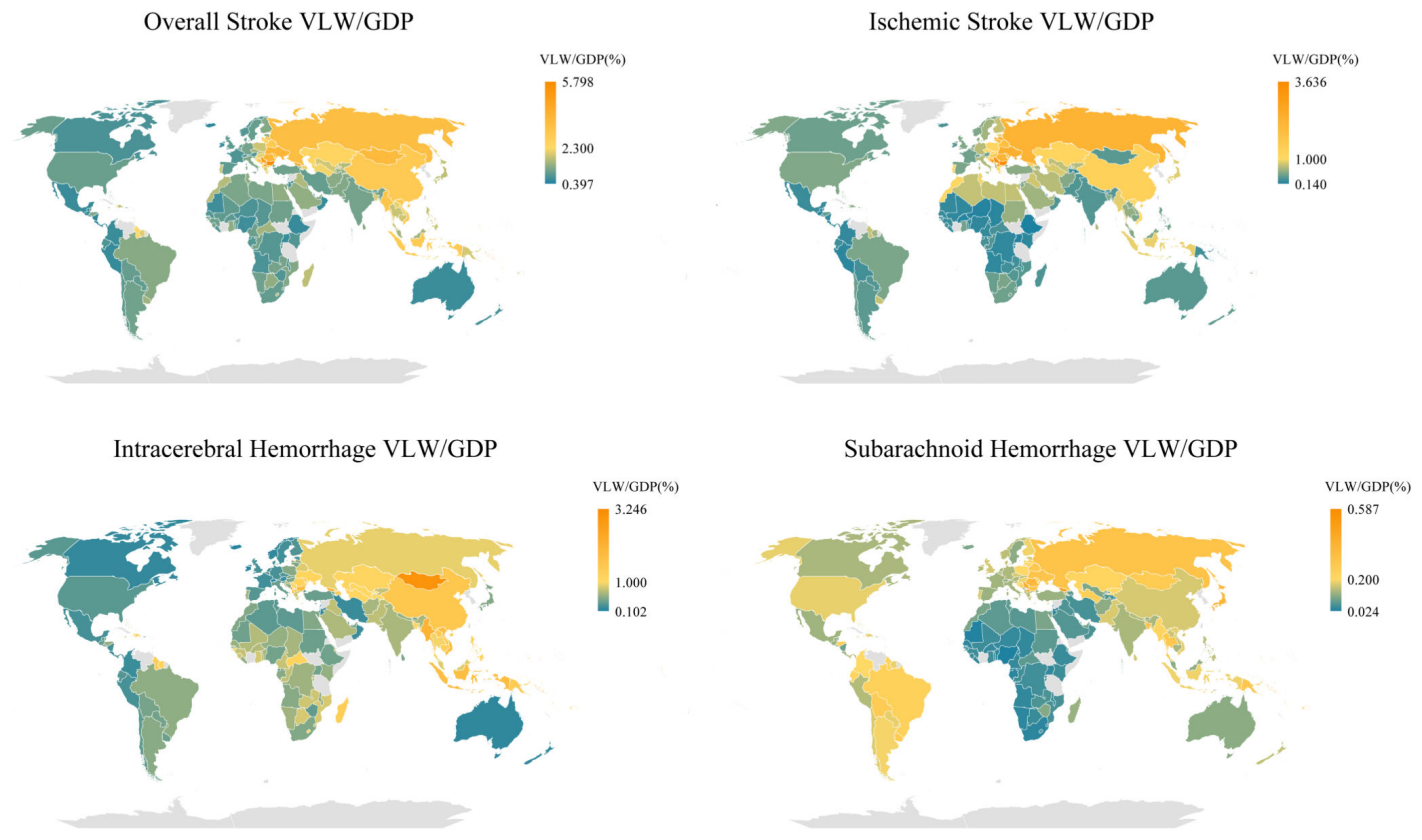
World heat maps of VLW/GDP in 2019 by country for stroke overall, ischemic stroke, intracerebral hemorrhage, and subarachnoid hemorrhage. VLW = Value of Lost Welfare, GDP = Gross Domestic Product.

The nationwide distribution for stroke overall and subtypes (i.e., ischemic stroke, intracerebral hemorrhage, and subarachnoid hemorrhage) in 2019 are illustrated in Figure 2 with the specific values presented in Table 1 and Supplementary Figure 1. The national composition of each GBD super-region is presented in Supplemental tables 2 and 3. Country-by-country estimates of VLW and VLW/GDP for stroke overall and for stroke subtypes in 2019 using IEs of 0.55 and 1.5 are presented in Supplemental Table 2 and Supplemental Table 3, respectively.

## Discussion

Consistent with prior epidemiological work,^1,7^ our study highlights the vast burden of disease associated with stroke, with the authors estimating global macroeconomic losses of over 2 trillion USD in 2019. It is the authors’ contention that the global, regional, and country-by-country estimates are of value for location-specific resource allocation, primary prevention, and priority setting.

The super-regions with the highest overall stroke VLW/GDP were 1) Central Europe, Eastern Europe, and Central Asia, and 2) Southeast Asia, East Asia, and Oceania. The High-Income super-region had a lower VLW/GDP than the global average for overall stroke and for all subtypes. These data add to a growing body of literature demonstrating a disproportionate stroke burden in LMICs.^29–32^ Critically, these regions lag in several aspects of stroke care that range from prevention and surveillance activities (e.g., the presence of registries, execution of recent risk factors surveys, and/or participation in research),^30^ to diagnosis/acute care and stroke units,^29–31^ to rehabilitation,^30,32^ all of which contribute to a greater disease burden and subsequent economic losses.

The proportion of global economic consequences made up by each stroke subtype was also consistent with previous GBD literature with ischemic stroke having accounted for the largest proportion, followed by intracerebral hemorrhage and subarachnoid hemorrhage.^1,7^ However, the economic consequences of stroke subtypes differed greatly according to super-region. For example, VLW/GDP due to subarachnoid hemorrhage was the third highest in the High-Income super-region which was largely comparable to LMIC super-regions. With regards to intracerebral hemorrhage, however, the High-Income super-region had the lowest VLW/GDP (0.33%) of the super-regions; with the Southeast Asian, East Asian and Oceanian super-region reporting a VLW/GDP of 1.48%. These patterns are consistent with evidence of increased risk of intracerebral hemorrhage in a subset LMICs possibly due to population-attributable risk of comorbidities such as hypertension.^33^

To further inform effective resource allocation, the authors recommend increased efforts to further subdivide stroke categories to the extent feasible. One pertinent example is that of ischemic stroke which had macroeconomic losses of over $100 billion in the United States alone. Amongst ischemic stroke, large vessel occlusion (LVO) is estimated to make up ~ 1/3 of cases.^34^ However, precise country-wide estimates of LVO as compared to non-LVO disease burden in other settings are lacking. Given that treatment with endovascular thrombectomy applies only to LVO and that untreated LVO is clinically devastating, such estimates, in both DALYs and macroeconomic terms, would be valuable when guiding the design of regional stroke systems and the allocation of finite resources.

In the 2016 GBD study on stroke, it was revealed that 87.9% of ischemic stroke DALYs and 89.5% of hemorrhagic stroke DALYs were due to potentially modifiable risk factors.^7^ It follows that there is a vast potential to decrease stroke disease burden by reducing risk factor exposure; for example, for every 1 USD spent on stroke and cardiovascular disease prevention, there is a 10.9 USD return on investment.^35^ Future efforts must focus on optimizing risk factors such as diabetes and hypertension, reducing tobacco use, and improving diet and physical activity.^36–40^ Despite this, the WHO estimates that implementation of specific population-wide interventions, in terms of current health spending, amounts to only 4% in low-income countries, 2% in lower middle-income countries, and less than 1% in upper middle-income countries.^40^ The WHO has further highlighted that effective response for prevention and control of noncommunicable disease requires contributions from various stakeholders including individuals, intergovernmental organizations, religious institutions, civil society, academia, the media, policy-makers, and industry.^40^ Finally, we recommend future assessments of cost-effectiveness of interventions aimed at addressing specific stroke subtypes.

While important we note that this study has several key limitations. First, it relies on modeling rather than empirical data. As an example, VSL estimates in individual countries are modelled using empirical US data and our transformation may not fully reflect regional variations. In an effort to minimize assumptions about willingness to pay after adjustment for economic size and purchasing power, an IE of 1.0 was ultimately selected. However, if country specific willingness to pay assumptions are available to a reader, a supplemental analysis using IEs of 0.55 and 1.5 is provided in Supplemental Table 2 and Supplemental Table 3, respectively. To maximize accuracy of VLW in a given region, further empirical regional estimates of VSL are needed and recommended by the authors. Second, the f(a) function which age adjusts VSL, does so according to Aldy and Viscusi’s estimates,^21^ these may also not reflect regional variations, limiting the accuracy of this analysis. Third, the DALY data provided by the GBD study is also largely modelled given the absence of high-quality epidemiological stroke studies in most countries. Finally, although the GBD study 2019 presents epidemiological data for three stroke sub-types for the first time, the authors would recommend further subclassification to the extent possible. The authors also recommend modelling of post-pandemic years once that GBD data is available.

The present study represents the first comprehensive report of macroeconomic losses associated with stroke and stroke sub-types on a global, regional, and country-by-country basis. Given the vast economic consequences of stroke and the availability of cost-effective interventions, it is the authors’ hope that these data may help justify increased spending on stroke, particularly in LMIC regions where the macroeconomic consequences of stroke are the highest.

## Supplementary Material

Graphical Abstract

Supplemental Publication Material

## Figures and Tables

**Table 1:** VLW and VLW/GDP by country in 2019 for stroke overall, ischemic stroke, intracerebral hemorrhage, and subarachnoid hemorrhage, generated using income elasticity (IE) of the VSL at 1.00. All $ values are in 2017 USD, PPP.

IE = 1.00	Stroke Overall		Intracerebral Hemorrahage		Ischemic Stroke		Subarachnoid Hemorrahage	
Country	VLW ($ billion)	VLW/GDP (%)	VLW ($ billion)	VLW/GDP (%)	VLW ($ billion)	VLW/GDP (%)	VLW ($ billion)	VLW/GDP (%)
Afghanistan	1.02	1.30	0.51	0.66	0.42	0.54	0.09	0.11
Albania	1.03	2.64	0.71	1.82	0.27	0.68	0.05	0.14
Algeria	5.94	1.20	1.74	0.35	3.83	0.77	0.37	0.07
Angola	1.66	0.78	1.12	0.53	0.45	0.21	0.08	0.04
Antigua and Barbuda	0.03	1.26	0.01	0.65	0.009	0.45	0.003	0.16
Argentina	10.92	1.10	5.27	0.53	3.74	0.38	1.90	0.19
Armenia	0.61	1.51	0.19	0.48	0.35	0.88	0.06	0.16
Australia	8.24	0.66	2.22	0.18	4.64	0.37	1.39	0.11
Austria	4.14	0.84	1.05	0.21	2.41	0.49	0.68	0.14
Azerbaijan	3.08	2.13	2.02	1.40	0.94	0.65	0.11	0.08
Bahamas	0.19	1.31	0.11	0.75	0.06	0.40	0.02	0.17
Bahrain	0.39	0.53	0.15	0.21	0.19	0.25	0.05	0.07
Bangladesh	15.29	1.97	8.73	1.13	4.11	0.53	2.45	0.32
Barbados	0.08	1.84	0.03	0.75	0.04	0.88	0.010	0.22
Belarus	5.44	2.99	1.58	0.87	3.35	1.84	0.51	0.28
Belgium	6.00	1.01	1.98	0.33	3.21	0.54	0.81	0.14
Belize	0.02	0.84	0.01	0.48	0.007	0.25	0.003	0.11
Benin	0.36	0.93	0.25	0.66	0.09	0.24	0.02	0.04
Bermuda	0.06	1.09	0.02	0.37	0.03	0.58	0.008	0.14
Bhutan	0.08	0.87	0.04	0.45	0.03	0.30	0.01	0.12
Bolivia	1.02	1.01	0.46	0.45	0.29	0.29	0.27	0.27
Bosnia and Herzegovina	1.65	3.35	0.27	0.54	1.26	2.56	0.12	0.25
Botswana	0.56	1.38	0.34	0.84	0.20	0.49	0.02	0.05
Brazil	40.33	1.29	17.33	0.56	15.52	0.50	7.48	0.24
Brunei	0.26	0.96	0.11	0.41	0.09	0.32	0.06	0.23
Bulgaria	9.38	5.80	3.01	1.86	5.88	3.64	0.48	0.30
Burkina Faso	0.41	0.92	0.31	0.70	0.08	0.18	0.02	0.04
Burundi	0.09	1.00	0.06	0.73	0.02	0.21	0.005	0.06
Cape Verde	0.05	1.19	0.03	0.78	0.01	0.37	0.001	0.04
Cambodia	1.38	1.91	0.92	1.27	0.37	0.51	0.10	0.13
Cameroon	1.00	1.06	0.74	0.78	0.22	0.24	0.04	0.04
Canada	14.24	0.77	3.60	0.20	8.19	0.44	2.46	0.13
Central African Republic	0.07	1.52	0.05	1.15	0.01	0.30	0.003	0.07
Chad	0.22	0.88	0.16	0.64	0.05	0.19	0.01	0.04
Chile	4.65	0.98	1.74	0.37	2.09	0.44	0.81	0.17
China	665.69	2.96	338.44	1.50	289.34	1.29	37.91	0.17
Colombia	4.81	0.65	1.66	0.23	1.68	0.23	1.48	0.20
Comoros	0.03	1.08	0.02	0.66	0.009	0.36	0.002	0.06
Congo	0.24	1.15	0.16	0.76	0.07	0.34	0.01	0.05
Costa Rica	0.56	0.55	0.21	0.20	0.23	0.23	0.13	0.12
Croatia	2.93	2.51	0.79	0.68	1.86	1.59	0.28	0.24
Cyprus	0.42	1.19	0.14	0.40	0.22	0.61	0.06	0.17
Czech Republic	6.76	1.55	1.39	0.32	4.63	1.06	0.74	0.17
Democratic Republic of the Congo	0.84	0.88	0.59	0.61	0.20	0.21	0.05	0.05
Denmark	3.44	1.04	1.00	0.30	1.94	0.58	0.51	0.15
Djibouti	0.07	1.34	0.05	0.85	0.02	0.40	0.005	0.09
Dominican Republic	3.33	1.69	1.91	0.96	1.08	0.55	0.35	0.18
Ecuador	1.62	0.82	0.63	0.32	0.54	0.27	0.45	0.23
Egypt	16.12	1.37	5.93	0.50	9.06	0.77	1.14	0.10
El Salvador	0.36	0.64	0.17	0.29	0.13	0.23	0.07	0.12
Equatorial Guinea	0.14	0.58	0.09	0.35	0.05	0.20	0.006	0.03
Estonia	0.66	1.37	0.14	0.29	0.44	0.92	0.08	0.17
Eswatini	0.11	1.14	0.07	0.72	0.04	0.38	0.004	0.04
Ethiopia	1.55	0.62	1.08	0.44	0.35	0.14	0.11	0.05
Fiji	0.23	1.86	0.12	0.96	0.07	0.59	0.04	0.31
Finland	3.42	1.27	0.82	0.31	2.09	0.78	0.50	0.19
France	25.63	0.83	7.53	0.24	14.12	0.46	3.98	0.13
Gabon	0.28	0.86	0.17	0.53	0.10	0.30	0.01	0.04
Gambia	0.05	0.93	0.03	0.63	0.01	0.26	0.002	0.04
Georgia	2.34	4.19	1.17	2.09	0.89	1.60	0.28	0.50
Germany	50.75	1.13	12.17	0.27	32.47	0.73	6.10	0.14
Ghana	2.22	1.35	1.36	0.83	0.78	0.47	0.08	0.05
Greece	6.50	2.04	2.54	0.80	3.51	1.10	0.46	0.14
Grenada	0.03	1.55	0.01	0.72	0.01	0.66	0.003	0.17
Guatemala	0.99	0.69	0.56	0.39	0.25	0.18	0.17	0.12
Guinea	0.35	1.06	0.25	0.77	0.08	0.25	0.02	0.05
Guinea-Bissau	0.05	1.25	0.03	0.93	0.01	0.27	0.002	0.05
Guyana	0.24	2.35	0.14	1.34	0.08	0.78	0.02	0.23
Haiti	0.71	2.16	0.41	1.25	0.18	0.56	0.12	0.35
Honduras	0.69	1.24	0.33	0.58	0.23	0.40	0.14	0.25
Hungary	6.72	2.11	1.56	0.49	4.55	1.43	0.60	0.19
Iceland	0.11	0.56	0.03	0.14	0.06	0.32	0.02	0.10
India	104.67	1.14	60.74	0.66	31.60	0.34	12.34	0.13
Indonesia	92.07	2.88	56.64	1.77	29.41	0.92	6.02	0.19
Iran	10.28	1.00	2.23	0.22	7.46	0.73	0.58	0.06
Iraq	6.92	1.63	3.18	0.75	3.47	0.82	0.27	0.06
Ireland	2.84	0.66	0.69	0.16	1.54	0.36	0.60	0.14
Israel	1.97	0.54	0.75	0.21	0.98	0.27	0.24	0.07
Italy	31.30	1.23	10.15	0.40	17.82	0.70	3.33	0.13
Jamaica	0.46	1.59	0.23	0.80	0.17	0.60	0.05	0.19
Japan	91.71	1.75	26.60	0.51	46.04	0.88	19.06	0.36
Jordan	0.80	0.79	0.23	0.23	0.53	0.52	0.04	0.04
Kazakhstan	12.27	2.52	5.40	1.11	5.78	1.18	1.10	0.22
Kenya	1.92	0.84	1.32	0.58	0.49	0.21	0.11	0.05
Kiribati	0.01	4.41	0.008	3.11	0.002	0.71	0.002	0.59
Kuwait	1.36	0.65	0.50	0.24	0.75	0.36	0.11	0.05
Kyrgyzstan	0.57	1.68	0.26	0.77	0.24	0.71	0.07	0.20
Laos	1.20	2.13	0.81	1.42	0.31	0.54	0.09	0.16
Latvia	1.94	3.28	0.36	0.62	1.44	2.44	0.13	0.22
Lebanon	0.53	0.53	0.10	0.10	0.40	0.40	0.02	0.02
Lesotho	0.10	1.67	0.07	1.17	0.03	0.45	0.003	0.06
Liberia	0.06	0.84	0.04	0.59	0.01	0.21	0.003	0.04
Libya	1.23	1.20	0.37	0.36	0.79	0.77	0.07	0.07
Lithuania	2.56	2.48	0.49	0.47	1.85	1.79	0.22	0.21
Luxembourg	0.53	0.75	0.18	0.25	0.28	0.40	0.07	0.10
Madagascar	0.76	1.75	0.57	1.30	0.14	0.33	0.05	0.12
Malawi	0.17	0.83	0.11	0.53	0.05	0.25	0.01	0.05
Malaysia	13.88	1.53	7.92	0.87	4.93	0.54	1.03	0.11
Maldives	0.08	0.78	0.05	0.44	0.03	0.26	0.008	0.08
Mali	0.44	0.97	0.32	0.70	0.10	0.22	0.02	0.05
Malta	0.19	0.86	0.06	0.27	0.11	0.50	0.02	0.08
Mauritania	0.16	0.69	0.10	0.43	0.05	0.23	0.007	0.03
Mauritius	0.53	1.83	0.25	0.86	0.21	0.73	0.07	0.24
Mexico	16.17	0.64	6.57	0.26	6.45	0.26	3.15	0.13
Federated States of Micronesia	0.01	2.80	0.008	2.00	0.002	0.49	0.001	0.31
Mongolia	1.45	3.66	1.20	3.02	0.15	0.37	0.11	0.27
Montenegro	0.57	4.26	0.43	3.25	0.12	0.90	0.02	0.12
Morocco	4.70	1.68	1.40	0.50	3.03	1.08	0.27	0.10
Mozambique	0.48	1.24	0.33	0.84	0.14	0.35	0.02	0.06
Myanmar	8.81	3.21	5.74	2.09	2.46	0.90	0.60	0.22
Namibia	0.27	1.10	0.15	0.63	0.11	0.43	0.009	0.04
Nepal	1.04	1.06	0.58	0.59	0.32	0.32	0.14	0.14
Netherlands	9.39	0.95	2.58	0.26	5.50	0.56	1.31	0.13
New Zealand	1.60	0.75	0.40	0.19	0.87	0.41	0.33	0.15
Nicaragua	0.21	0.58	0.09	0.26	0.08	0.23	0.03	0.09
Niger	0.23	0.79	0.17	0.58	0.05	0.17	0.01	0.04
Nigeria	7.10	0.69	4.64	0.45	2.20	0.21	0.27	0.03
Republic of North Macedonia	1.72	4.97	0.51	1.49	1.05	3.05	0.15	0.43
Norway	2.97	0.86	0.67	0.20	1.79	0.52	0.51	0.15
Oman	0.85	0.63	0.32	0.23	0.45	0.33	0.08	0.06
Pakistan	12.74	1.25	7.52	0.74	3.48	0.34	1.74	0.17
Panama	1.01	0.75	0.43	0.33	0.38	0.28	0.19	0.14
Papua New Guinea	0.74	1.94	0.53	1.38	0.11	0.28	0.11	0.28
Paraguay	0.87	0.98	0.40	0.45	0.31	0.35	0.16	0.18
Peru	2.58	0.62	1.13	0.27	0.86	0.21	0.59	0.14
Philippines	17.60	1.83	11.67	1.21	4.52	0.47	1.41	0.15
Poland	23.51	1.87	6.59	0.52	14.34	1.14	2.57	0.20
Portugal	6.68	1.86	2.20	0.61	3.87	1.08	0.62	0.17
Puerto Rico	1.01	0.91	0.40	0.36	0.47	0.42	0.13	0.12
Qatar	1.01	0.40	0.41	0.16	0.40	0.16	0.21	0.08
Moldova	1.26	3.64	0.51	1.48	0.68	1.96	0.07	0.19
Romania	21.90	3.79	6.86	1.19	12.98	2.24	2.05	0.36
Russia	139.20	3.49	36.36	0.91	90.49	2.27	12.36	0.31
Rwanda	0.28	0.99	0.20	0.71	0.06	0.21	0.02	0.06
Saint Lucia	0.05	1.70	0.02	0.83	0.02	0.70	0.005	0.18
Saint Vincent and the Grenadines	0.02	1.80	0.01	0.94	0.010	0.72	0.002	0.15
Samoa	0.03	2.40	0.02	1.59	0.007	0.54	0.003	0.26
São Tomé and Príncipe	0.01	1.25	0.007	0.83	0.003	0.38	0.000	0.05
Saudi Arabia	22.26	1.38	10.94	0.68	10.26	0.64	1.06	0.07
Senegal	0.48	0.87	0.33	0.59	0.13	0.24	0.02	0.04
Serbia	6.37	5.01	1.35	1.06	4.51	3.55	0.51	0.40
Seychelles	0.05	1.80	0.03	1.01	0.02	0.69	0.003	0.11
Sierra Leone	0.15	1.15	0.11	0.80	0.04	0.30	0.007	0.05
Singapore	3.97	0.71	1.47	0.26	1.82	0.33	0.68	0.12
Slovakia	3.18	1.83	0.84	0.49	2.07	1.19	0.26	0.15
Slovenia	1.07	1.32	0.28	0.35	0.67	0.82	0.12	0.15
Solomon Islands	0.07	4.03	0.06	3.12	0.010	0.53	0.007	0.38
South Africa	7.27	0.99	3.69	0.51	3.31	0.45	0.26	0.04
Spain	18.27	0.95	6.30	0.33	9.61	0.50	2.36	0.12
Sri Lanka	3.76	1.32	1.34	0.47	1.95	0.68	0.47	0.17
Sudan	1.97	1.10	0.74	0.42	1.09	0.61	0.13	0.07
Suriname	0.20	2.03	0.12	1.21	0.06	0.64	0.02	0.18
Sweden	5.54	1.02	1.40	0.26	3.53	0.65	0.61	0.11
Switzerland	4.08	0.70	0.94	0.16	2.47	0.42	0.68	0.12
Tajikistan	0.45	1.40	0.36	1.13	0.07	0.22	0.02	0.06
Thailand	23.68	1.84	12.35	0.96	7.60	0.59	3.72	0.29
Timor-Leste	0.09	1.94	0.06	1.22	0.03	0.59	0.006	0.13
Togo	0.13	1.04	0.09	0.73	0.03	0.26	0.006	0.04
Tonga	0.008	1.18	0.004	0.65	0.003	0.39	0.001	0.13
Trinidad and Tobago	0.55	1.53	0.22	0.60	0.26	0.73	0.07	0.19
Tunisia	1.72	1.37	0.44	0.35	1.18	0.94	0.10	0.08
Turkey	25.71	1.09	9.64	0.41	13.19	0.56	2.87	0.12
Turkmenistan	2.04	2.20	1.01	1.09	0.80	0.86	0.23	0.25
Uganda	0.64	0.66	0.44	0.45	0.16	0.17	0.04	0.04
Ukraine	20.66	3.84	6.05	1.12	13.01	2.42	1.61	0.30
United Arab Emirates	8.39	1.28	3.23	0.49	4.58	0.70	0.58	0.09
United Kingdom	29.67	0.96	8.40	0.27	16.11	0.52	5.16	0.17
United States	214.61	1.04	70.55	0.34	106.67	0.52	37.39	0.18
Uruguay	1.09	1.48	0.31	0.42	0.58	0.79	0.20	0.27
Uzbekistan	4.52	1.92	2.49	1.06	1.79	0.76	0.24	0.10
Vanuatu	0.03	3.01	0.02	2.07	0.006	0.61	0.003	0.33
Vietnam	23.50	3.03	14.19	1.83	7.92	1.02	1.40	0.18
Zambia	0.71	1.14	0.50	0.81	0.17	0.27	0.04	0.06
Zimbabwe	0.34	0.84	0.16	0.38	0.14	0.35	0.04	0.11
