# Supplementary figures and images for: The global, regional, and national economic consequences of stroke
Source: Stroke. Author manuscript; Available in PMC 2023 Sep 1. (PMC7614992; doi:10.1161/STROKEAHA.123.043131)

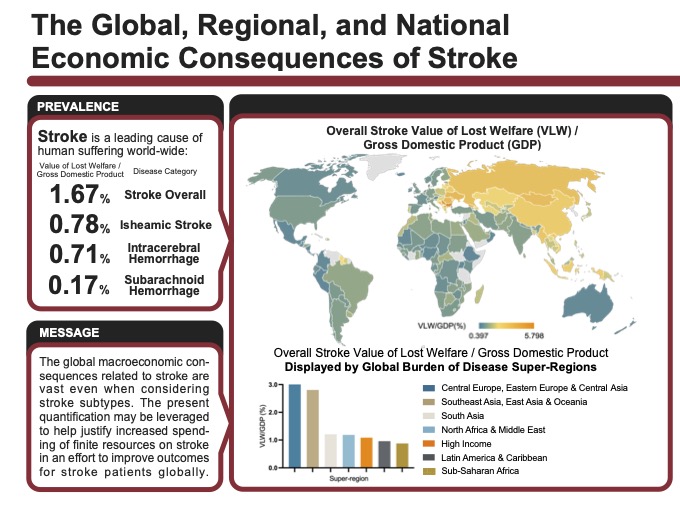

Supplement: Graphical Abstract [file EMS178545-supplement-Graphical_Abstract.jpg]
