# Supplementary material for: The global, regional, and national economic consequences of stroke
Source: Stroke. Author manuscript; Available in PMC 2023 Sep 1. (PMC7614992; doi:10.1161/STROKEAHA.123.043131)
Supplement: Supplemental Publication Material [file EMS178545-supplement-Supplemental_Publication_Material.pdf]

SUPPLEMENTAL MATERIAL

Supplemental Table 1. ICD-10 codes included in the Global Burden of Disease study definition of stroke.

**I60**	**Subarachnoid hemorrhage**
I60.0	Subarachnoid hemorrhage from carotid siphon and bifurcation
I60.1	Subarachnoid hemorrhage from middle cerebral artery
I60.2	Subarachnoid hemorrhage from anterior communicating artery
I60.3	Subarachnoid hemorrhage from posterior communicating artery
I60.4	Subarachnoid hemorrhage from basilar artery
I60.5	Subarachnoid hemorrhage from vertebral artery
I60.6	Subarachnoid hemorrhage from other intracranial arteries
I60.7	Subarachnoid hemorrhage from intracranial artery, unspecified
I60.8	Other subarachnoid hemorrhage
I60.9	Subarachnoid hemorrhage, unspecified
**I61**	**Intracerebral hemorrhage**
I61.0	Intracerebral hemorrhage in hemisphere, subcortical
I61.1	Intracerebral hemorrhage in hemisphere, cortical
I61.2	Intracerebral hemorrhage in hemisphere, unspecified
I61.3	Intracerebral hemorrhage in brain stem
I61.4	Intracerebral hemorrhage in cerebellum
I61.5	Intracerebral hemorrhage, intraventricular
I61.6	Intracerebral hemorrhage, multiple localized
I61.8	Other intracerebral hemorrhage
I61.9	Intracerebral hemorrhage, unspecified
**I63**	**Cerebral infarction**
I63.0	Cerebral infarction due to thrombosis of precerebral arteries
I63.1	Cerebral infarction due to embolism of precerebral arteries
I63.2	Cerebral infarction due to unspecified occlusion or stenosis of precerebral arteries
I63.3	Cerebral infarction due to thrombosis of cerebral arteries
I63.4	Cerebral infarction due to embolism of cerebral arteries
I63.5	Cerebral infarction due to unspecified occlusion or stenosis of cerebral arteries
I63.6	Cerebral infarction due to cerebral venous thrombosis, nonpyogenic
I63.8	Other cerebral infarction
I63.9	Cerebral infarction, unspecified
**I64**	**Stroke, not specified as hemorrhage or infarction**
**I69**	**Sequelae of cerebrovascular disease**
I69.0	Sequelae of subarachnoid hemorrhage
I69.1	Sequelae of intracerebral hemorrhage
I69.3	Sequelae of cerebral infarction

Value of Lost Welfare by Country

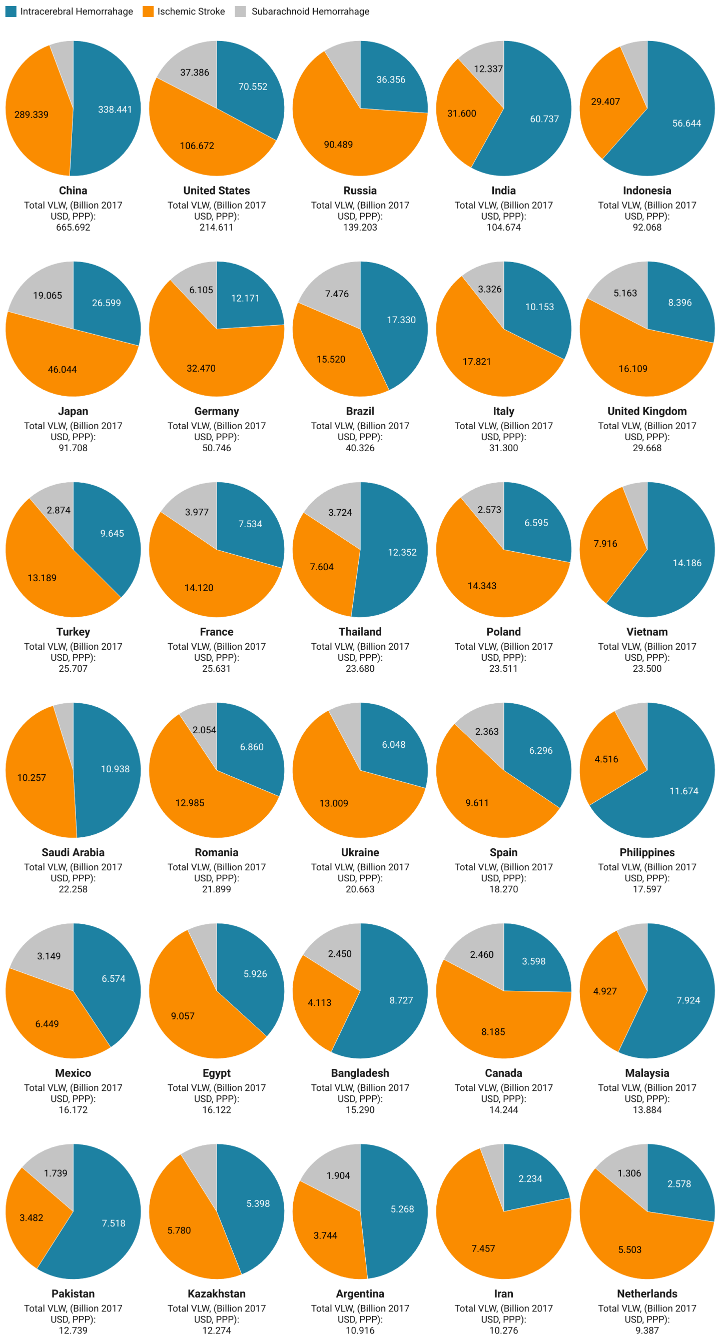

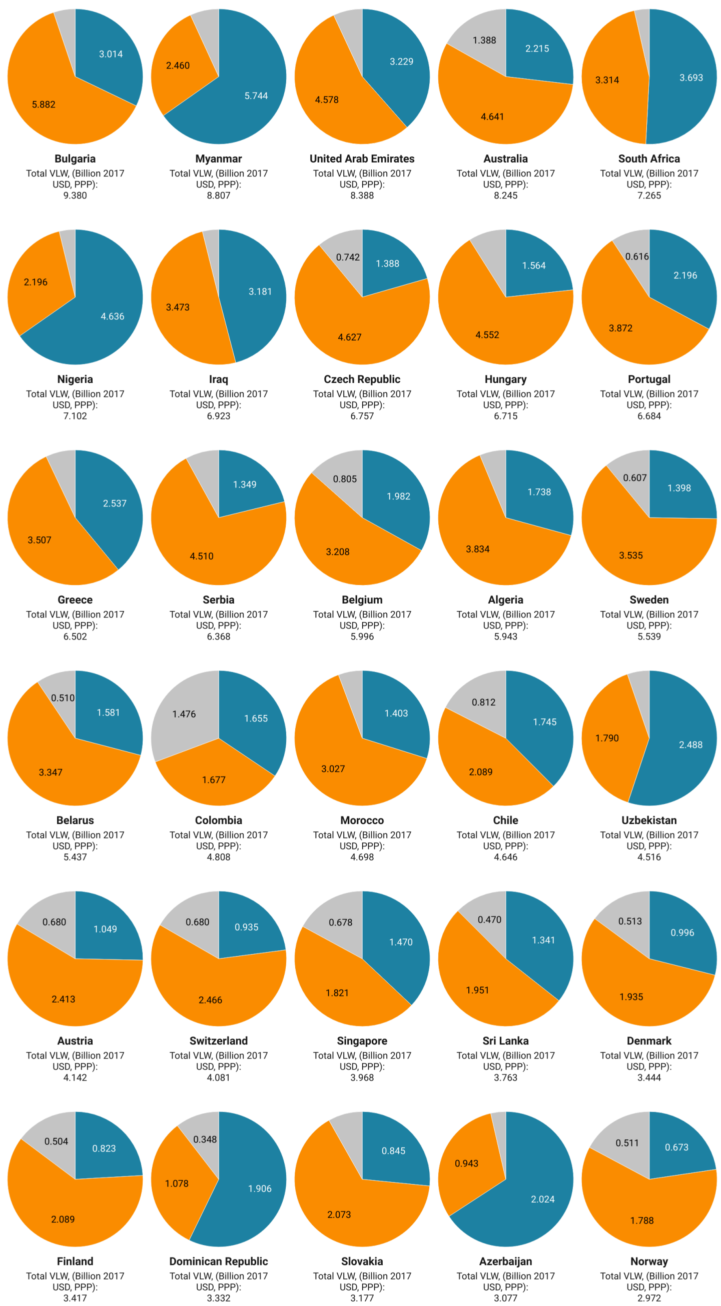
3.014
5.882
Bulgaria
Total VLW, (Billion 2017 USD, PPP): 9.380
2.460
5.744
Myanmar
Total VLW, (Billion 2017 USD, PPP): 8.807
3.229
4.578
United Arab Emirates
Total VLW, (Billion 2017 USD, PPP): 8.388
1.388
2.215
4.641
Australia
Total VLW, (Billion 2017 USD, PPP): 8.245
3.314
3.693
South Africa
Total VLW, (Billion 2017 USD, PPP): 7.265
2.196
4.636
Nigeria
Total VLW, (Billion 2017 USD, PPP): 7.102
3.181
3.473
Iraq
Total VLW, (Billion 2017 USD, PPP): 6.923
0.742
1.388
4.627
Czech Republic
Total VLW, (Billion 2017 USD, PPP): 6.757
1.564
4.552
Hungary
Total VLW, (Billion 2017 USD, PPP): 6.715
0.616
2.196
3.872
Portugal
Total VLW, (Billion 2017 USD, PPP): 6.684
2.537
3.507
Greece
Total VLW, (Billion 2017 USD, PPP): 6.502
1.349
4.510
Serbia
Total VLW, (Billion 2017 USD, PPP): 6.368
0.805
1.982
3.208
Belgium
Total VLW, (Billion 2017 USD, PPP): 5.996
1.738
3.834
Algeria
Total VLW, (Billion 2017 USD, PPP): 5.943
0.607
1.398
3.535
Sweden
Total VLW, (Billion 2017 USD, PPP): 5.539
0.510
1.581
3.347
Belarus
Total VLW, (Billion 2017 USD, PPP): 5.437
1.476
1.655
1.677
Colombia
Total VLW, (Billion 2017 USD, PPP): 4.808
1.403
3.027
Morocco
Total VLW, (Billion 2017 USD, PPP): 4.698
0.812
1.745
2.089
Chile
Total VLW, (Billion 2017 USD, PPP): 4.646
1.790
2.488
Uzbekistan
Total VLW, (Billion 2017 USD, PPP): 4.516
0.680
1.049
2.413
Austria
Total VLW, (Billion 2017 USD, PPP): 4.142
0.680
0.935
2.466
Switzerland
Total VLW, (Billion 2017 USD, PPP): 4.081
0.678
1.470
1.821
Singapore
Total VLW, (Billion 2017 USD, PPP): 3.968
0.470
1.341
1.951
Sri Lanka
Total VLW, (Billion 2017 USD, PPP): 3.763
0.513
0.996
1.935
Denmark
Total VLW, (Billion 2017 USD, PPP): 3.444
0.504
0.823
2.089
Finland
Total VLW, (Billion 2017 USD, PPP): 3.417
0.348
1.078
1.906
Dominican Republic
Total VLW, (Billion 2017 USD, PPP): 3.332
0.845
2.073
Slovakia
Total VLW, (Billion 2017 USD, PPP): 3.177
0.943
2.024
Azerbaijan
Total VLW, (Billion 2017 USD, PPP): 3.077
0.511
0.673
1.788
Norway
Total VLW, (Billion 2017 USD, PPP): 2.972

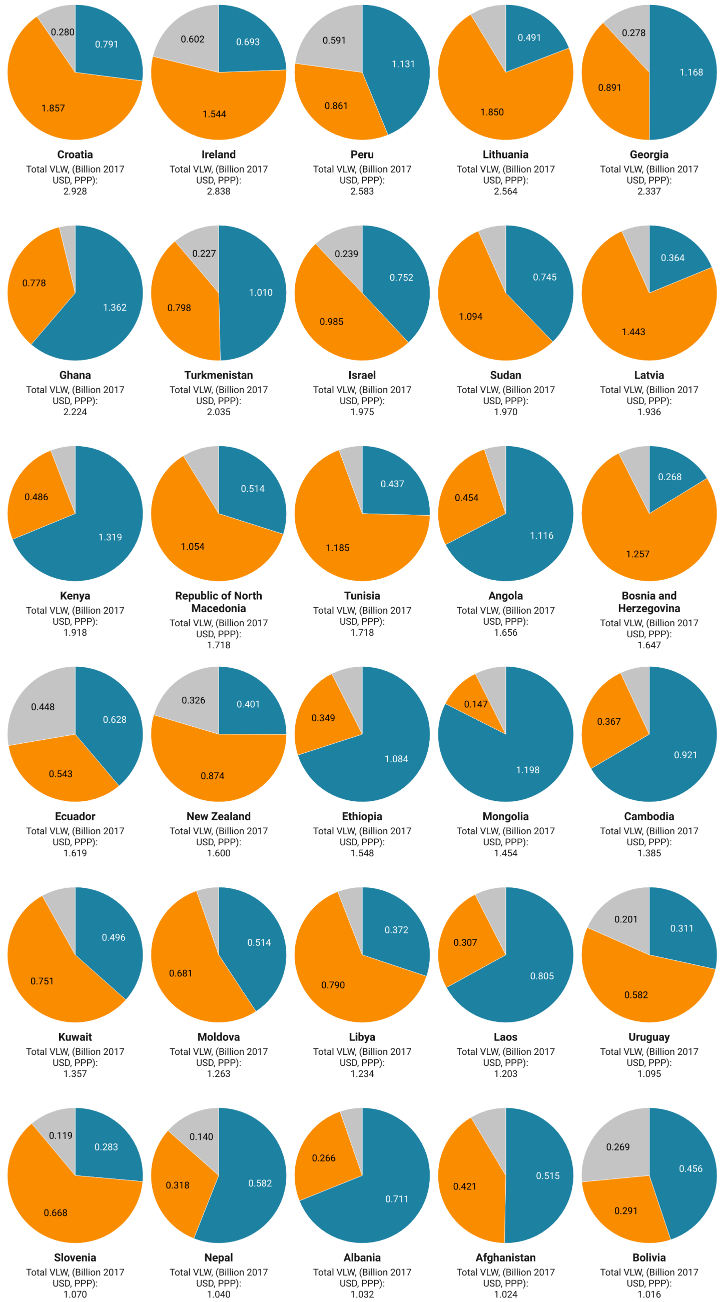
0.280
0.791
1.857
Croatia
Total VLW, (Billion 2017 USD, PPP): 2.928
0.602
0.693
1.544
Ireland
Total VLW, (Billion 2017 USD, PPP): 2.838
0.591
1.131
0.861
Peru
Total VLW, (Billion 2017 USD, PPP): 2.583
0.491
1.850
Lithuania
Total VLW, (Billion 2017 USD, PPP): 2.564
0.278
1.168
0.891
Georgia
Total VLW, (Billion 2017 USD, PPP): 2.337
0.778
1.362
Ghana
Total VLW, (Billion 2017 USD, PPP): 2.224
0.227
1.010
0.798
Turkmenistan
Total VLW, (Billion 2017 USD, PPP): 2.035
0.239
0.752
0.985
Israel
Total VLW, (Billion 2017 USD, PPP): 1.975
0.745
1.094
Sudan
Total VLW, (Billion 2017 USD, PPP): 1.970
0.364
1.443
Latvia
Total VLW, (Billion 2017 USD, PPP): 1.936
0.486
1.319
Kenya
Total VLW, (Billion 2017 USD, PPP): 1.918
0.514
1.054
Republic of North Macedonia
Total VLW, (Billion 2017 USD, PPP): 1.718
0.437
1.185
Tunisia
Total VLW, (Billion 2017 USD, PPP): 1.718
0.454
1.116
Angola
Total VLW, (Billion 2017 USD, PPP): 1.656
0.268
1.257
Bosnia and Herzegovina
Total VLW, (Billion 2017 USD, PPP): 1.647
0.448
0.628
0.543
Ecuador
Total VLW, (Billion 2017 USD, PPP): 1.619
0.326
0.401
0.874
New Zealand
Total VLW, (Billion 2017 USD, PPP): 1.600
0.349
1.084
Ethiopia
Total VLW, (Billion 2017 USD, PPP): 1.548
0.147
1.198
Mongolia
Total VLW, (Billion 2017 USD, PPP): 1.454
0.367
0.921
Cambodia
Total VLW, (Billion 2017 USD, PPP): 1.385
0.496
0.751
Kuwait
Total VLW, (Billion 2017 USD, PPP): 1.357
0.514
0.681
Moldova
Total VLW, (Billion 2017 USD, PPP): 1.263
0.372
0.790
Libya
Total VLW, (Billion 2017 USD, PPP): 1.234
0.307
0.805
Laos
Total VLW, (Billion 2017 USD, PPP): 1.203
0.201
0.311
0.582
Uruguay
Total VLW, (Billion 2017 USD, PPP): 1.095
0.119
0.283
0.668
Slovenia
Total VLW, (Billion 2017 USD, PPP): 1.070
0.140
0.318
0.582
Nepal
Total VLW, (Billion 2017 USD, PPP): 1.040
0.266
0.711
Albania
Total VLW, (Billion 2017 USD, PPP): 1.032
0.421
0.515
Afghanistan
Total VLW, (Billion 2017 USD, PPP): 1.024
0.269
0.456
0.291
Bolivia
Total VLW, (Billion 2017 USD, PPP): 1.016

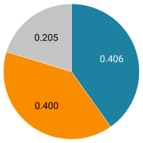

Qatar
Total VLW, (Billion 2017 USD, PPP): 1.012

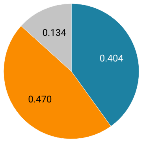

Puerto Rico
Total VLW, (Billion 2017 USD, PPP): 1.008

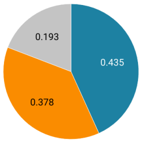

Panama
Total VLW, (Billion 2017 USD, PPP): 1.006

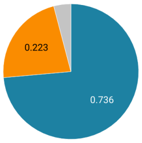

Cameroon
Total VLW, (Billion 2017 USD, PPP): 1.001

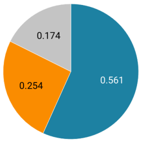

Guatemala
Total VLW, (Billion 2017 USD, PPP): 0.989

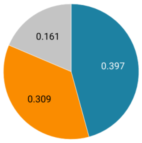

Paraguay
Total VLW, (Billion 2017 USD, PPP): 0.868

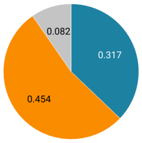

Oman
Total VLW, (Billion 2017 USD, PPP): 0.853

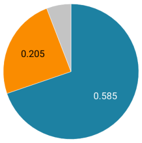

Democratic Republic of the Congo
Total VLW, (Billion 2017 USD, PPP): 0.839

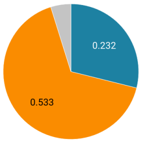

Jordan
Total VLW, (Billion 2017 USD, PPP): 0.804

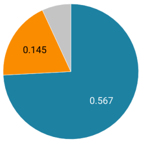

Madagascar
Total VLW, (Billion 2017 USD, PPP): 0.765

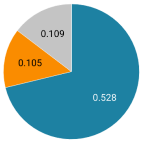

Papua New Guinea
Total VLW, (Billion 2017 USD, PPP): 0.742

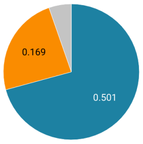

Zambia
Total VLW, (Billion 2017 USD, PPP): 0.708

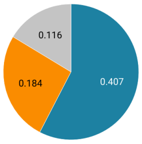

Haiti
Total VLW, (Billion 2017 USD, PPP): 0.707

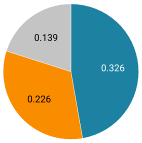

Honduras
Total VLW, (Billion 2017 USD, PPP): 0.691

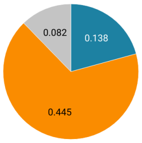

Estonia
Total VLW, (Billion 2017 USD, PPP): 0.664

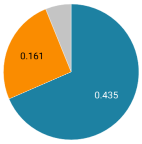

Uganda
Total VLW, (Billion 2017 USD, PPP): 0.636

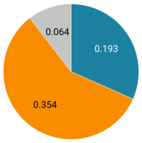

Armenia
Total VLW, (Billion 2017 USD, PPP): 0.611

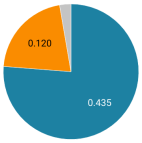

Montenegro
Total VLW, (Billion 2017 USD, PPP): 0.570

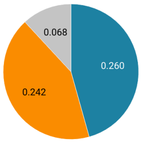

Kyrgyzstan
Total VLW, (Billion 2017 USD, PPP): 0.570

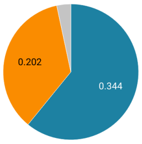

Botswana
Total VLW, (Billion 2017 USD, PPP): 0.564

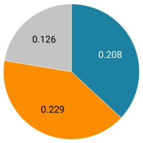

Costa Rica
Total VLW, (Billion 2017 USD, PPP): 0.562

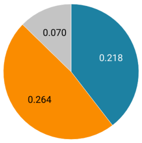

Trinidad and Tobago
Total VLW, (Billion 2017 USD, PPP): 0.552

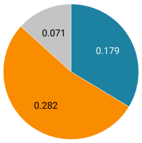

Luxembourg
Total VLW, (Billion 2017 USD, PPP): 0.531

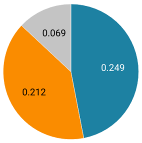

Mauritius
Total VLW, (Billion 2017 USD, PPP): 0.530

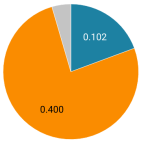

Lebanon
Total VLW, (Billion 2017 USD, PPP): 0.526

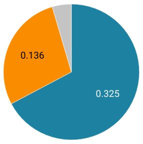

Mozambique
Total VLW, (Billion 2017 USD, PPP): 0.484

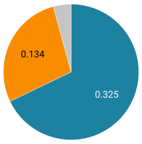

Senegal
Total VLW, (Billion 2017 USD, PPP): 0.479

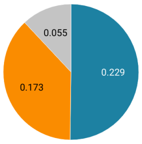

Jamaica
Total VLW, (Billion 2017 USD, PPP): 0.457

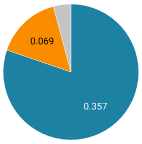

Tajikistan
Total VLW, (Billion 2017 USD, PPP): 0.445

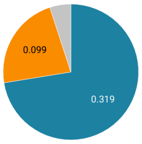

Mali
Total VLW, (Billion 2017 USD, PPP): 0.441

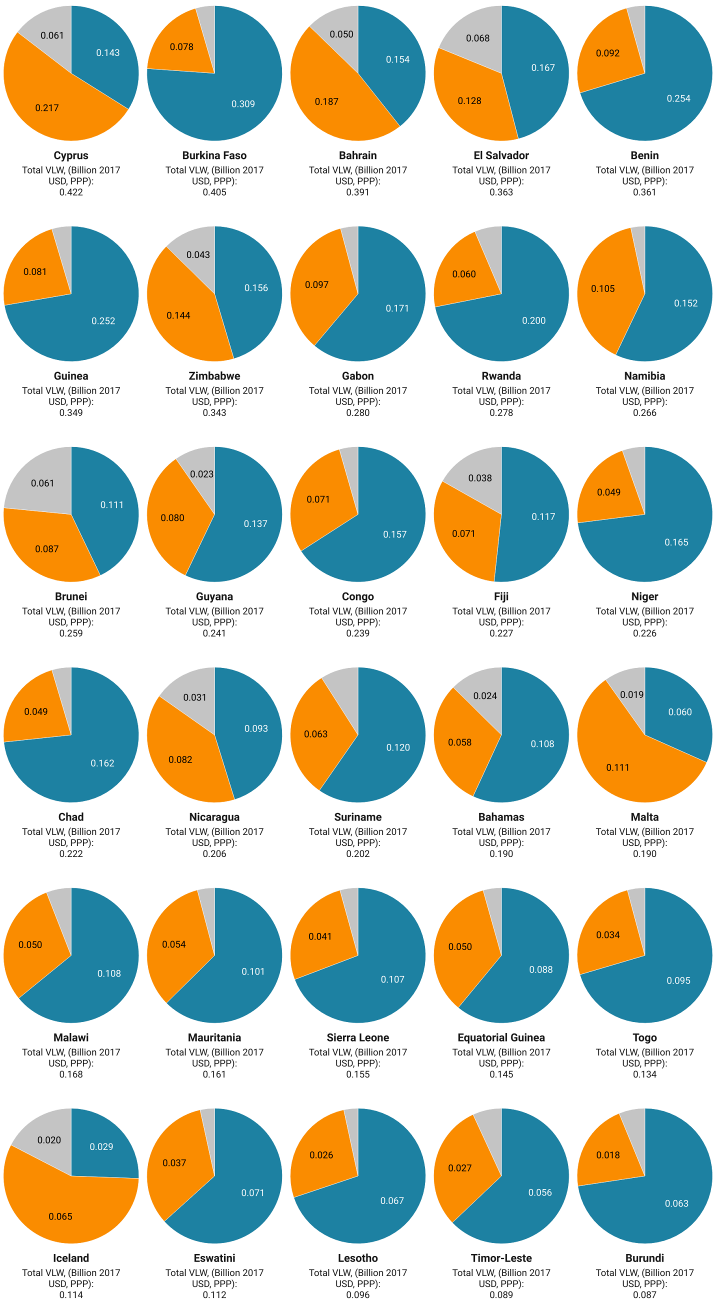

0.061
0.143
0.217
Cyprus
Total VLW, (Billion 2017 USD, PPP): 0.422
0.078
0.309
Burkina Faso
Total VLW, (Billion 2017 USD, PPP): 0.405
0.050
0.154
0.187
Bahrain
Total VLW, (Billion 2017 USD, PPP): 0.391
0.068
0.167
0.128
El Salvador
Total VLW, (Billion 2017 USD, PPP): 0.363
0.092
0.254
Benin
Total VLW, (Billion 2017 USD, PPP): 0.361
0.081
0.252
Guinea
Total VLW, (Billion 2017 USD, PPP): 0.349
0.043
0.156
0.144
Zimbabwe
Total VLW, (Billion 2017 USD, PPP): 0.343
0.097
0.171
Gabon
Total VLW, (Billion 2017 USD, PPP): 0.280
0.060
0.200
Rwanda
Total VLW, (Billion 2017 USD, PPP): 0.278
0.105
0.152
Namibia
Total VLW, (Billion 2017 USD, PPP): 0.266
0.061
0.111
0.087
Brunei
Total VLW, (Billion 2017 USD, PPP): 0.259
0.023
0.080
0.137
Guyana
Total VLW, (Billion 2017 USD, PPP): 0.241
0.071
0.157
Congo
Total VLW, (Billion 2017 USD, PPP): 0.239
0.038
0.117
0.071
Fiji
Total VLW, (Billion 2017 USD, PPP): 0.227
0.049
0.165
Niger
Total VLW, (Billion 2017 USD, PPP): 0.226
0.049
0.162
Chad
Total VLW, (Billion 2017 USD, PPP): 0.222
0.031
0.093
0.082
Nicaragua
Total VLW, (Billion 2017 USD, PPP): 0.206
0.063
0.120
Suriname
Total VLW, (Billion 2017 USD, PPP): 0.202
0.024
0.058
0.108
Bahamas
Total VLW, (Billion 2017 USD, PPP): 0.190
0.019
0.060
0.111
Malta
Total VLW, (Billion 2017 USD, PPP): 0.190
0.050
0.108
Malawi
Total VLW, (Billion 2017 USD, PPP): 0.168
0.054
0.101
Mauritania
Total VLW, (Billion 2017 USD, PPP): 0.161
0.041
0.107
Sierra Leone
Total VLW, (Billion 2017 USD, PPP): 0.155
0.050
0.088
Equatorial Guinea
Total VLW, (Billion 2017 USD, PPP): 0.145
0.034
0.095
Togo
Total VLW, (Billion 2017 USD, PPP): 0.134
0.020
0.029
0.065
Iceland
Total VLW, (Billion 2017 USD, PPP): 0.114
0.037
0.071
Eswatini
Total VLW, (Billion 2017 USD, PPP): 0.112
0.026
0.067
Lesotho
Total VLW, (Billion 2017 USD, PPP): 0.096
0.027
0.056
Timor-Leste
Total VLW, (Billion 2017 USD, PPP): 0.089
0.018
0.063
Burundi
Total VLW, (Billion 2017 USD, PPP): 0.087

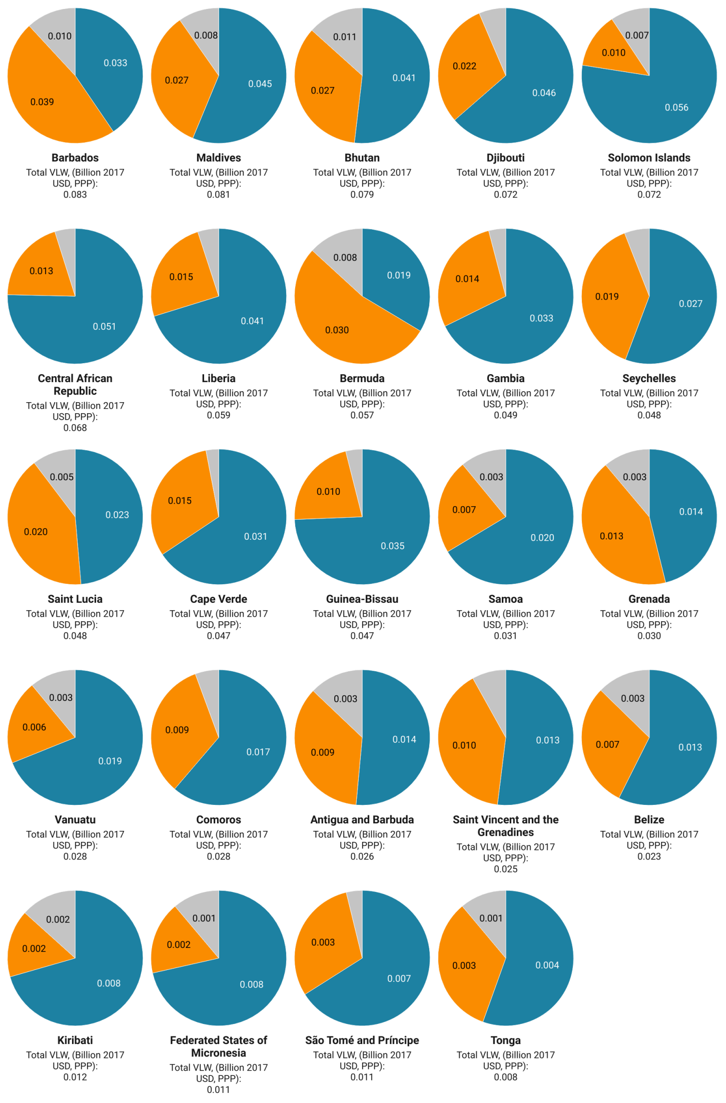

Supplementary Figure 1. Pie charts for individual countries displaying the relative proportion of stroke subtypes (ischemic stroke, intracerebral hemorrhage and subarachnoid hemorrhage) as a share of total stroke country VLW (2017 USD, PPP).

Supplemental Table 2. VLW and VLW/GDP in 2019 for stroke overall, ischemic stroke, intracerebral hemorrhage, and subarachnoid hemorrhage, generated using income elasticity (IE) at 0.55. All $ values are in 2017 USD, PPP.

IE = 0.55		Stroke Overall		Intracerebral Hemorrahage		Ischemic Stroke		Subarachnoid Hemorrahage	
Country	Region	VLW ($ billion)	VLW/GDP (%)	VLW ($ billion)	VLW/GDP (%)	VLW ($ billion)	VLW/GDP (%)	VLW ($ billion)	VLW/GDP (%)
Afghanistan	North Africa & Middle East	4.75	6.05	2.39	3.04	1.95	2.49	0.41	0.52
Albania	Central Europe, Eastern Europe & Central Asia	2.05	5.24	1.41	3.61	0.53	1.35	0.11	0.28
Algeria	North Africa & Middle East	12.73	2.57	3.72	0.75	8.21	1.66	0.79	0.16
Angola	Sub-Saharan Africa	4.53	2.14	3.06	1.44	1.24	0.59	0.23	0.11
Antigua and Barbuda	Latin America & Caribbean	0.04	2.03	0.02	1.05	0.02	0.72	0.005	0.26
Argentina	High Income	17.45	1.76	8.42	0.85	5.98	0.60	3.04	0.31
Armenia	Central Europe, Eastern Europe & Central Asia	1.21	3.00	0.38	0.95	0.70	1.74	0.13	0.31
Australia	High Income	9.16	0.73	2.46	0.20	5.16	0.41	1.54	0.12
Austria	High Income	4.36	0.88	1.10	0.22	2.54	0.51	0.72	0.14
Azerbaijan	Central Europe, Eastern Europe & Central Asia	5.95	4.11	3.92	2.70	1.82	1.26	0.21	0.15
Bahamas	Latin America & Caribbean	0.24	1.66	0.14	0.95	0.07	0.51	0.03	0.21
Bahrain	North Africa & Middle East	0.45	0.61	0.18	0.24	0.22	0.29	0.06	0.08
Bangladesh	South Asia	48.76	6.29	27.83	3.59	13.12	1.69	7.81	1.01
Barbados	Latin America & Caribbean	0.15	3.44	0.06	1.39	0.07	1.64	0.02	0.41
Belarus	Central Europe, Eastern Europe & Central Asia	9.23	5.08	2.69	1.48	5.68	3.13	0.87	0.48
Belgium	High Income	6.53	1.10	2.16	0.36	3.49	0.59	0.88	0.15
Belize	Latin America & Caribbean	0.06	2.22	0.04	1.28	0.02	0.66	0.008	0.28
Benin	Sub-Saharan Africa	1.36	3.51	0.96	2.47	0.34	0.89	0.06	0.15
Bermuda	Latin America & Caribbean	0.05	0.97	0.02	0.33	0.03	0.52	0.007	0.13
Bhutan	South Asia	0.17	1.85	0.09	0.96	0.06	0.64	0.02	0.25
Bolivia	Latin America & Caribbean	2.47	2.45	1.11	1.10	0.71	0.70	0.65	0.65
Bosnia and Herzegovina	Central Europe, Eastern Europe & Central Asia	3.14	6.39	0.51	1.04	2.40	4.87	0.23	0.48
Botswana	Sub-Saharan Africa	0.99	2.43	0.61	1.48	0.36	0.87	0.03	0.08
Brazil	Latin America & Caribbean	77.24	2.48	33.19	1.07	29.73	0.95	14.32	0.46
Brunei	High Income	0.26	0.97	0.11	0.41	0.09	0.33	0.06	0.23
Bulgaria	Central Europe, Eastern Europe & Central Asia	14.66	9.06	4.71	2.91	9.19	5.68	0.76	0.47
Burkina Faso	Sub-Saharan Africa	1.84	4.15	1.40	3.16	0.36	0.80	0.08	0.19
Burundi	Sub-Saharan Africa	0.64	7.34	0.46	5.33	0.13	1.55	0.04	0.46

Cape Verde	Sub-Saharan Africa	0.12	3.16	0.08	2.08	0.04	0.99	0.004	0.10
Cambodia	Southeast Asia, East Asia, & Oceania	4.58	6.33	3.04	4.21	1.21	1.68	0.32	0.44
Cameroon	Sub-Saharan Africa	3.60	3.82	2.65	2.81	0.80	0.85	0.15	0.16
Canada	High Income	15.90	0.86	4.02	0.22	9.13	0.50	2.75	0.15
Central African Republic	Sub-Saharan Africa	0.45	10.03	0.34	7.56	0.09	1.98	0.02	0.49
Chad	Sub-Saharan Africa	1.16	4.61	0.85	3.38	0.26	1.02	0.05	0.21
Chile	High Income	7.02	1.48	2.64	0.56	3.16	0.67	1.23	0.26
China	Southeast Asia, East Asia, & Oceania	1226.35	5.45	623.48	2.77	533.02	2.37	69.84	0.31
Colombia	Latin America & Caribbean	9.26	1.26	3.19	0.43	3.23	0.44	2.84	0.39
Comoros	Sub-Saharan Africa	0.11	4.20	0.07	2.57	0.04	1.39	0.006	0.24
Congo	Sub-Saharan Africa	0.84	4.01	0.55	2.64	0.25	1.19	0.04	0.18
Costa Rica	Latin America & Caribbean	0.94	0.92	0.35	0.34	0.38	0.38	0.21	0.21
Croatia	Central Europe, Eastern Europe & Central Asia	4.15	3.55	1.12	0.96	2.64	2.25	0.40	0.34
Cyprus	High Income	0.51	1.45	0.17	0.49	0.26	0.75	0.07	0.21
Czech Republic	Central Europe, Eastern Europe & Central Asia	8.17	1.87	1.68	0.38	5.60	1.28	0.90	0.21
Democratic Republic of the Congo	Sub-Saharan Africa	5.17	5.43	3.61	3.79	1.26	1.33	0.30	0.32
Denmark	High Income	3.59	1.08	1.04	0.31	2.02	0.61	0.53	0.16
Djibouti	Sub-Saharan Africa	0.22	3.99	0.14	2.54	0.06	1.20	0.01	0.26
Dominican Republic	Latin America & Caribbean	5.78	2.92	3.30	1.67	1.87	0.95	0.60	0.31
Ecuador	Latin America & Caribbean	3.49	1.77	1.35	0.68	1.17	0.59	0.97	0.49
Egypt	North Africa & Middle East	34.20	2.90	12.57	1.06	19.21	1.63	2.42	0.20
El Salvador	Latin America & Caribbean	0.88	1.55	0.40	0.71	0.31	0.55	0.17	0.29
Equatorial Guinea	Sub-Saharan Africa	0.25	1.00	0.15	0.61	0.09	0.35	0.01	0.04
Estonia	Central Europe, Eastern Europe & Central Asia	0.85	1.75	0.18	0.36	0.57	1.17	0.10	0.21
Eswatini	Sub-Saharan Africa	0.27	2.77	0.17	1.75	0.09	0.92	0.009	0.09
Ethiopia	Sub-Saharan Africa	6.95	2.79	4.87	1.95	1.57	0.63	0.52	0.21
Fiji	Southeast Asia, East Asia, & Oceania	0.45	3.69	0.23	1.91	0.14	1.16	0.08	0.62
Finland	High Income	3.83	1.43	0.92	0.34	2.34	0.87	0.56	0.21
France	High Income	29.43	0.95	8.65	0.28	16.21	0.52	4.57	0.15
Gabon	Sub-Saharan Africa	0.53	1.64	0.33	1.00	0.19	0.57	0.02	0.07
Gambia	Sub-Saharan Africa	0.22	4.19	0.15	2.83	0.06	1.18	0.009	0.17

Georgia	Central Europe, Eastern Europe & Central Asia	4.45	7.97	2.22	3.98	1.70	3.04	0.53	0.95
Germany	High Income	54.30	1.21	13.02	0.29	34.75	0.78	6.53	0.15
Ghana	Sub-Saharan Africa	6.69	4.06	4.10	2.49	2.34	1.42	0.25	0.15
Greece	High Income	9.09	2.85	3.55	1.11	4.90	1.54	0.64	0.20
Grenada	Latin America & Caribbean	0.05	2.79	0.02	1.28	0.02	1.19	0.006	0.31
Guatemala	Latin America & Caribbean	2.41	1.68	1.37	0.95	0.62	0.43	0.42	0.30
Guinea	Sub-Saharan Africa	1.47	4.48	1.06	3.24	0.34	1.03	0.07	0.21
Guinea-Bissau	Sub-Saharan Africa	0.22	5.99	0.17	4.45	0.05	1.30	0.009	0.24
Guyana	Latin America & Caribbean	0.49	4.75	0.28	2.71	0.16	1.58	0.05	0.46
Haiti	Latin America & Caribbean	2.81	8.60	1.62	4.96	0.73	2.24	0.46	1.41
Honduras	Latin America & Caribbean	2.03	3.62	0.96	1.71	0.66	1.18	0.41	0.73
Hungary	Central Europe, Eastern Europe & Central Asia	9.01	2.83	2.10	0.66	6.11	1.92	0.80	0.25
Iceland	High Income	0.12	0.59	0.03	0.15	0.07	0.33	0.02	0.10
India	South Asia	285.71	3.11	165.78	1.81	86.25	0.94	33.68	0.37
Indonesia	Southeast Asia, East Asia, & Oceania	194.93	6.10	119.93	3.75	62.26	1.95	12.74	0.40
Iran	North Africa & Middle East	21.29	2.07	4.63	0.45	15.45	1.50	1.21	0.12
Iraq	North Africa & Middle East	15.25	3.59	7.01	1.65	7.65	1.80	0.59	0.14
Ireland	High Income	2.45	0.57	0.60	0.14	1.33	0.31	0.52	0.12
Israel	High Income	2.41	0.67	0.92	0.25	1.20	0.33	0.29	0.08
Italy	High Income	37.18	1.46	12.06	0.47	21.17	0.83	3.95	0.16
Jamaica	Latin America & Caribbean	1.05	3.65	0.53	1.83	0.40	1.38	0.13	0.44
Japan	High Income	110.33	2.11	32.00	0.61	55.40	1.06	22.94	0.44
Jordan	North Africa & Middle East	1.83	1.80	0.53	0.52	1.21	1.19	0.09	0.09
Kazakhstan	Central Europe, Eastern Europe & Central Asia	18.11	3.71	7.97	1.63	8.53	1.75	1.62	0.33
Kenya	Sub-Saharan Africa	6.38	2.80	4.39	1.93	1.62	0.71	0.38	0.16
Kiribati	Southeast Asia, East Asia, & Oceania	0.05	19.62	0.04	13.84	0.008	3.17	0.007	2.61
Kuwait	North Africa & Middle East	1.50	0.72	0.55	0.26	0.83	0.40	0.12	0.06
Kyrgyzstan	Central Europe, Eastern Europe & Central Asia	1.74	5.11	0.79	2.33	0.74	2.17	0.21	0.61
Laos	Southeast Asia, East Asia, & Oceania	3.05	5.40	2.05	3.62	0.78	1.38	0.23	0.40
Latvia	Central Europe, Eastern Europe & Central Asia	2.66	4.51	0.50	0.85	1.98	3.36	0.18	0.30
Lebanon	North Africa & Middle East	1.01	1.02	0.20	0.20	0.77	0.77	0.05	0.05
Lesotho	Sub-Saharan Africa	0.39	6.90	0.28	4.82	0.11	1.85	0.01	0.23

Liberia	Sub-Saharan Africa	0.32	4.59	0.23	3.22	0.08	1.14	0.02	0.23
Libya	North Africa & Middle East	2.34	2.27	0.70	0.68	1.49	1.45	0.14	0.13
Lithuania	Central Europe, Eastern Europe & Central Asia	3.24	3.13	0.62	0.60	2.34	2.26	0.28	0.27
Luxembourg	High Income	0.41	0.57	0.14	0.19	0.22	0.30	0.05	0.08
Madagascar	Sub-Saharan Africa	3.96	9.08	2.94	6.73	0.75	1.72	0.27	0.63
Malawi	Sub-Saharan Africa	1.04	5.16	0.67	3.31	0.31	1.55	0.06	0.31
Malaysia	Southeast Asia, East Asia, & Oceania	19.82	2.19	11.31	1.25	7.03	0.78	1.48	0.16
Maldives	Southeast Asia, East Asia, & Oceania	0.14	1.31	0.08	0.74	0.05	0.45	0.01	0.13
Mali	Sub-Saharan Africa	1.94	4.25	1.41	3.08	0.44	0.96	0.10	0.21
Malta	High Income	0.22	1.01	0.07	0.32	0.13	0.59	0.02	0.10
Mauritania	Sub-Saharan Africa	0.49	2.10	0.31	1.31	0.16	0.70	0.02	0.09
Mauritius	Southeast Asia, East Asia, & Oceania	0.83	2.88	0.39	1.35	0.33	1.15	0.11	0.38
Mexico	Latin America & Caribbean	27.20	1.08	11.06	0.44	10.85	0.43	5.30	0.21
Federated States of Micronesia	Southeast Asia, East Asia, & Oceania	0.04	10.29	0.03	7.35	0.007	1.79	0.005	1.15
Mongolia	Central Europe, Eastern Europe & Central Asia	3.02	7.60	2.49	6.27	0.31	0.77	0.22	0.56
Montenegro	Central Europe, Eastern Europe & Central Asia	0.92	6.88	0.70	5.24	0.19	1.45	0.02	0.19
Morocco	North Africa & Middle East	12.17	4.36	3.64	1.30	7.84	2.81	0.70	0.25
Mozambique	Sub-Saharan Africa	2.78	7.15	1.87	4.81	0.78	2.02	0.13	0.33
Myanmar	Southeast Asia, East Asia, & Oceania	27.25	9.92	17.77	6.47	7.61	2.77	1.87	0.68
Namibia	Sub-Saharan Africa	0.61	2.53	0.35	1.45	0.24	1.00	0.02	0.08
Nepal	South Asia	3.84	3.91	2.15	2.19	1.17	1.19	0.52	0.53
Netherlands	High Income	9.80	1.00	2.69	0.27	5.75	0.58	1.36	0.14
New Zealand	High Income	1.90	0.89	0.47	0.22	1.04	0.48	0.39	0.18
Nicaragua	Latin America & Caribbean	0.62	1.73	0.28	0.78	0.24	0.69	0.09	0.26
Niger	Sub-Saharan Africa	1.33	4.65	0.97	3.39	0.29	1.00	0.07	0.25
Nigeria	Sub-Saharan Africa	21.87	2.12	14.28	1.38	6.76	0.66	0.83	0.08
Republic of North Macedonia	Central Europe, Eastern Europe & Central Asia	3.12	9.03	0.93	2.70	1.92	5.54	0.27	0.79
Norway	High Income	2.93	0.85	0.66	0.19	1.76	0.51	0.50	0.15
Oman	North Africa & Middle East	1.24	0.91	0.46	0.34	0.66	0.49	0.12	0.09
Pakistan	South Asia	40.87	4.02	24.12	2.37	11.17	1.10	5.58	0.55
Panama	Latin America & Caribbean	1.37	1.03	0.59	0.44	0.52	0.39	0.26	0.20

Papua New Guinea	Southeast Asia, East Asia, & Oceania	2.46	6.45	1.75	4.59	0.35	0.91	0.36	0.94
Paraguay	Latin America & Caribbean	1.78	2.01	0.82	0.92	0.64	0.72	0.33	0.37
Peru	Latin America & Caribbean	5.27	1.26	2.31	0.55	1.76	0.42	1.20	0.29
Philippines	Southeast Asia, East Asia, & Oceania	42.29	4.39	28.05	2.91	10.85	1.13	3.38	0.35
Poland	Central Europe, Eastern Europe & Central Asia	31.30	2.49	8.78	0.70	19.09	1.52	3.43	0.27
Portugal	High Income	8.69	2.42	2.86	0.80	5.04	1.40	0.80	0.22
Puerto Rico	Latin America & Caribbean	1.31	1.18	0.53	0.47	0.61	0.55	0.17	0.16
Qatar	North Africa & Middle East	0.86	0.34	0.35	0.14	0.34	0.13	0.17	0.07
Moldova	Central Europe, Eastern Europe & Central Asia	2.56	7.38	1.04	3.01	1.38	3.98	0.14	0.39
Romania	Central Europe, Eastern Europe & Central Asia	30.55	5.28	9.57	1.65	18.11	3.13	2.87	0.50
Russia	Central Europe, Eastern Europe & Central Asia	202.46	5.07	52.88	1.32	131.61	3.30	17.97	0.45
Rwanda	Sub-Saharan Africa	1.25	4.43	0.90	3.19	0.27	0.96	0.08	0.28
Saint Lucia	Latin America & Caribbean	0.09	3.18	0.04	1.55	0.04	1.31	0.009	0.33
Saint Vincent and the Grenadines	Latin America & Caribbean	0.05	3.72	0.03	1.93	0.02	1.49	0.004	0.30
Samoa	Southeast Asia, East Asia, & Oceania	0.09	6.64	0.06	4.41	0.02	1.51	0.009	0.73
São Tomé and Príncipe	Sub-Saharan Africa	0.04	4.31	0.02	2.84	0.01	1.30	0.001	0.16
Saudi Arabia	North Africa & Middle East	25.32	1.57	12.44	0.77	11.67	0.73	1.21	0.08
Senegal	Sub-Saharan Africa	1.78	3.26	1.21	2.21	0.50	0.91	0.08	0.14
Serbia	Central Europe, Eastern Europe & Central Asia	11.07	8.72	2.35	1.85	7.84	6.17	0.89	0.70
Seychelles	Southeast Asia, East Asia, & Oceania	0.07	2.61	0.04	1.46	0.03	1.00	0.004	0.16
Sierra Leone	Sub-Saharan Africa	0.78	5.80	0.54	4.01	0.21	1.54	0.03	0.25
Singapore	High Income	3.24	0.58	1.20	0.21	1.49	0.27	0.55	0.10
Slovakia	Central Europe, Eastern Europe & Central Asia	4.30	2.47	1.14	0.66	2.81	1.61	0.35	0.20
Slovenia	Central Europe, Eastern Europe & Central Asia	1.32	1.63	0.35	0.43	0.83	1.02	0.15	0.18
Solomon Islands	Southeast Asia, East Asia, & Oceania	0.30	16.69	0.23	12.93	0.04	2.21	0.03	1.55
South Africa	Sub-Saharan Africa	15.00	2.05	7.63	1.04	6.85	0.94	0.53	0.07
Spain	High Income	22.14	1.15	7.63	0.40	11.65	0.61	2.86	0.15
Sri Lanka	Southeast Asia, East Asia, & Oceania	7.61	2.67	2.71	0.95	3.95	1.39	0.95	0.33

Sudan	North Africa & Middle East	6.65	3.71	2.52	1.40	3.70	2.06	0.44	0.25
Suriname	Latin America & Caribbean	0.36	3.65	0.22	2.18	0.11	1.14	0.03	0.33
Sweden	High Income	5.98	1.10	1.51	0.28	3.81	0.70	0.65	0.12
Switzerland	High Income	3.92	0.67	0.90	0.15	2.37	0.40	0.65	0.11
Tajikistan	Central Europe, Eastern Europe & Central Asia	1.65	5.21	1.32	4.18	0.26	0.81	0.07	0.22
Thailand	Southeast Asia, East Asia, & Oceania	41.02	3.19	21.40	1.67	13.17	1.03	6.45	0.50
Timor-Leste	Southeast Asia, East Asia, & Oceania	0.32	7.04	0.20	4.43	0.10	2.13	0.02	0.48
Togo	Sub-Saharan Africa	0.70	5.41	0.49	3.81	0.18	1.37	0.03	0.22
Tonga	Southeast Asia, East Asia, & Oceania	0.02	3.29	0.01	1.82	0.007	1.10	0.002	0.37
Trinidad and Tobago	Latin America & Caribbean	0.82	2.27	0.32	0.90	0.39	1.08	0.10	0.29
Tunisia	North Africa & Middle East	3.79	3.02	0.96	0.77	2.62	2.08	0.21	0.17
Turkey	North Africa & Middle East	36.79	1.56	13.80	0.59	18.88	0.80	4.11	0.17
Turkmenistan	Central Europe, Eastern Europe & Central Asia	3.81	4.13	1.89	2.05	1.49	1.62	0.43	0.46
Uganda	Sub-Saharan Africa	2.87	2.97	1.97	2.03	0.73	0.75	0.18	0.18
Ukraine	Central Europe, Eastern Europe & Central Asia	42.18	7.84	12.35	2.29	26.56	4.93	3.28	0.61
United Arab Emirates	North Africa & Middle East	8.13	1.24	3.13	0.48	4.44	0.68	0.56	0.09
United Kingdom	High Income	33.93	1.09	9.60	0.31	18.43	0.59	5.91	0.19
United States	High Income	214.61	1.04	70.55	0.34	106.67	0.52	37.39	0.18
Uruguay	High Income	1.78	2.40	0.50	0.68	0.94	1.28	0.33	0.44
Uzbekistan	Central Europe, Eastern Europe & Central Asia	12.09	5.13	6.66	2.83	4.79	2.03	0.63	0.27
Vanuatu	Southeast Asia, East Asia, & Oceania	0.11	11.61	0.07	8.00	0.02	2.33	0.01	1.28
Vietnam	Southeast Asia, East Asia, & Oceania	59.16	7.63	35.71	4.60	19.93	2.57	3.52	0.45
Zambia	Sub-Saharan Africa	2.60	4.20	1.84	2.97	0.62	1.00	0.14	0.22
Zimbabwe	Sub-Saharan Africa	1.39	3.39	0.63	1.54	0.58	1.42	0.18	0.43

VLW = Value of Lost Welfare, GDP = Gross Domestic Product.

Supplemental Table 3. VLW and VLW/GDP in 2019 for stroke overall, ischemic stroke, intracerebral hemorrhage, and subarachnoid hemorrhage, generated using income elasticity (IE) at 1.50. All values are in 2017 USD, PPP.

IE = 1.50		Stroke Overall		Intracerebral Hemorrhage		Ischemic Stroke		Subarachnoid Hemorrhage	
Country	Region	VLW ($ billion)	VLW/GDP (%)	VLW ($ billion)	VLW/GDP (%)	VLW ($ billion)	VLW/GDP (%)	VLW ($ billion)	VLW/GDP (%)
Afghanistan	North Africa & Middle East	0.19	0.24	0.09	0.12	0.08	0.10	0.02	0.02
Albania	Central Europe, Eastern Europe & Central Asia	0.48	1.24	0.33	0.85	0.12	0.32	0.03	0.07
Algeria	North Africa & Middle East	2.55	0.51	0.75	0.15	1.64	0.33	0.16	0.03
Angola	Sub-Saharan Africa	0.54	0.25	0.36	0.17	0.15	0.07	0.03	0.01
Antigua and Barbuda	Latin America & Caribbean	0.02	0.74	0.008	0.38	0.006	0.26	0.002	0.10
Argentina	High Income	6.48	0.65	3.13	0.32	2.22	0.22	1.13	0.11
Armenia	Central Europe, Eastern Europe & Central Asia	0.29	0.71	0.09	0.22	0.17	0.41	0.03	0.07
Australia	High Income	7.33	0.58	1.97	0.16	4.13	0.33	1.23	0.10
Austria	High Income	3.91	0.79	0.99	0.20	2.28	0.46	0.64	0.13
Azerbaijan	Central Europe, Eastern Europe & Central Asia	1.48	1.02	0.97	0.67	0.45	0.31	0.05	0.04
Bahamas	Latin America & Caribbean	0.15	1.01	0.08	0.58	0.04	0.31	0.02	0.13
Bahrain	North Africa & Middle East	0.33	0.45	0.13	0.18	0.16	0.21	0.04	0.06
Bangladesh	South Asia	4.21	0.54	2.41	0.31	1.13	0.15	0.68	0.09
Barbados	Latin America & Caribbean	0.04	0.92	0.02	0.37	0.02	0.44	0.005	0.11
Belarus	Central Europe, Eastern Europe & Central Asia	3.02	1.66	0.88	0.48	1.86	1.02	0.28	0.16
Belgium	High Income	5.45	0.92	1.80	0.30	2.92	0.49	0.73	0.12
Belize	Latin America & Caribbean	0.008	0.28	0.005	0.16	0.002	0.08	0.001	0.04
Benin	Sub-Saharan Africa	0.08	0.21	0.06	0.15	0.02	0.05	0.004	0.009
Bermuda	Latin America & Caribbean	0.07	1.25	0.02	0.42	0.03	0.67	0.009	0.17
Bhutan	South Asia	0.03	0.38	0.02	0.20	0.01	0.13	0.005	0.05
Bolivia	Latin America & Caribbean	0.38	0.38	0.17	0.17	0.11	0.11	0.10	0.10
Bosnia and Herzegovina	Central Europe, Eastern Europe & Central Asia	0.80	1.63	0.13	0.27	0.61	1.25	0.06	0.12
Botswana	Sub-Saharan Africa	0.30	0.73	0.18	0.45	0.11	0.26	0.01	0.02
Brazil	Latin America & Caribbean	19.59	0.63	8.42	0.27	7.54	0.24	3.63	0.12
Brunei	High Income	0.26	0.96	0.11	0.41	0.09	0.32	0.06	0.22
Bulgaria	Central Europe, Eastern Europe & Central Asia	5.71	3.53	1.84	1.13	3.58	2.21	0.29	0.18
Burkina Faso	Sub-Saharan Africa	0.08	0.17	0.06	0.13	0.01	0.03	0.003	0.008
Burundi	Sub-Saharan Africa	0.010	0.11	0.007	0.08	0.002	0.02	0.001	0.007

Cape Verde	Sub-Saharan Africa	0.02	0.40	0.01	0.27	0.005	0.13	0.000	0.01
Cambodia	Southeast Asia, East Asia, & Oceania	0.37	0.51	0.24	0.34	0.10	0.13	0.03	0.04
Cameroon	Sub-Saharan Africa	0.24	0.26	0.18	0.19	0.05	0.06	0.010	0.01
Canada	High Income	12.61	0.68	3.19	0.17	7.25	0.39	2.18	0.12
Central African Republic	Sub-Saharan Africa	0.008	0.19	0.006	0.14	0.002	0.04	0.000	0.009
Chad	Sub-Saharan Africa	0.04	0.14	0.03	0.10	0.008	0.03	0.002	0.006
Chile	High Income	2.94	0.62	1.10	0.23	1.32	0.28	0.51	0.11
China	Southeast Asia, East Asia, & Oceania	337.64	1.50	171.66	0.76	146.75	0.65	19.23	0.09
Colombia	Latin America & Caribbean	2.32	0.32	0.80	0.11	0.81	0.11	0.71	0.10
Comoros	Sub-Saharan Africa	0.006	0.24	0.004	0.15	0.002	0.08	0.000	0.01
Congo	Sub-Saharan Africa	0.06	0.29	0.04	0.19	0.02	0.08	0.003	0.01
Costa Rica	Latin America & Caribbean	0.32	0.31	0.12	0.12	0.13	0.13	0.07	0.07
Croatia	Central Europe, Eastern Europe & Central Asia	1.99	1.70	0.54	0.46	1.26	1.08	0.19	0.16
Cyprus	High Income	0.34	0.95	0.11	0.32	0.17	0.49	0.05	0.14
Czech Republic	Central Europe, Eastern Europe & Central Asia	5.47	1.25	1.12	0.26	3.75	0.86	0.60	0.14
Democratic Republic of the Congo	Sub-Saharan Africa	0.11	0.12	0.08	0.08	0.03	0.03	0.007	0.007
Denmark	High Income	3.29	0.99	0.95	0.29	1.85	0.56	0.49	0.15
Djibouti	Sub-Saharan Africa	0.02	0.40	0.01	0.25	0.006	0.12	0.001	0.03
Dominican Republic	Latin America & Caribbean	1.81	0.91	1.03	0.52	0.58	0.30	0.19	0.10
Ecuador	Latin America & Caribbean	0.69	0.35	0.27	0.14	0.23	0.12	0.19	0.10
Egypt	North Africa & Middle East	6.99	0.59	2.57	0.22	3.93	0.33	0.49	0.04
El Salvador	Latin America & Caribbean	0.14	0.24	0.06	0.11	0.05	0.08	0.03	0.05
Equatorial Guinea	Sub-Saharan Africa	0.08	0.31	0.05	0.19	0.03	0.11	0.003	0.01
Estonia	Central Europe, Eastern Europe & Central Asia	0.51	1.05	0.11	0.22	0.34	0.70	0.06	0.13
Eswatini	Sub-Saharan Africa	0.04	0.42	0.03	0.27	0.01	0.14	0.001	0.01
Ethiopia	Sub-Saharan Africa	0.29	0.12	0.20	0.08	0.07	0.03	0.02	0.009
Fiji	Southeast Asia, East Asia, & Oceania	0.11	0.87	0.05	0.45	0.03	0.27	0.02	0.15
Finland	High Income	3.01	1.12	0.73	0.27	1.84	0.69	0.44	0.17
France	High Income	21.98	0.71	6.46	0.21	12.11	0.39	3.41	0.11
Gabon	Sub-Saharan Africa	0.14	0.42	0.08	0.26	0.05	0.15	0.006	0.02
Gambia	Sub-Saharan Africa	0.009	0.18	0.006	0.12	0.003	0.05	0.000	0.007

Georgia	Central Europe, Eastern Europe & Central Asia	1.14	2.05	0.57	1.03	0.44	0.78	0.14	0.24
Germany	High Income	47.06	1.05	11.29	0.25	30.11	0.67	5.66	0.13
Ghana	Sub-Saharan Africa	0.65	0.40	0.40	0.24	0.23	0.14	0.02	0.02
Greece	High Income	4.48	1.41	1.75	0.55	2.42	0.76	0.32	0.10
Grenada	Latin America & Caribbean	0.02	0.81	0.007	0.37	0.007	0.35	0.002	0.09
Guatemala	Latin America & Caribbean	0.37	0.26	0.21	0.15	0.09	0.07	0.06	0.05
Guinea	Sub-Saharan Africa	0.07	0.22	0.05	0.16	0.02	0.05	0.003	0.010
Guinea-Bissau	Sub-Saharan Africa	0.008	0.22	0.006	0.16	0.002	0.05	0.000	0.009
Guyana	Latin America & Caribbean	0.11	1.08	0.06	0.61	0.04	0.36	0.01	0.10
Haiti	Latin America & Caribbean	0.15	0.47	0.09	0.27	0.04	0.12	0.02	0.08
Honduras	Latin America & Caribbean	0.21	0.37	0.10	0.18	0.07	0.12	0.04	0.08
Hungary	Central Europe, Eastern Europe & Central Asia	4.84	1.52	1.13	0.35	3.28	1.03	0.43	0.14
Iceland	High Income	0.11	0.53	0.03	0.14	0.06	0.30	0.02	0.09
India	South Asia	34.30	0.37	19.90	0.22	10.35	0.11	4.04	0.04
Indonesia	Southeast Asia, East Asia, & Oceania	40.007	1.25	24.61	0.77	12.78	0.40	2.61	0.08
Iran	North Africa & Middle East	4.57	0.45	0.99	0.10	3.32	0.32	0.26	0.03
Iraq	North Africa & Middle East	2.88	0.68	1.32	0.31	1.44	0.34	0.11	0.03
Ireland	High Income	3.34	0.78	0.82	0.19	1.82	0.42	0.71	0.17
Israel	High Income	1.58	0.44	0.60	0.17	0.79	0.22	0.19	0.05
Italy	High Income	25.85	1.01	8.39	0.33	14.72	0.58	2.75	0.11
Jamaica	Latin America & Caribbean	0.18	0.63	0.09	0.31	0.07	0.24	0.02	0.08
Japan	High Income	74.68	1.43	21.66	0.41	37.49	0.72	15.52	0.30
Jordan	North Africa & Middle East	0.32	0.32	0.09	0.09	0.21	0.21	0.02	0.02
Kazakhstan	Central Europe, Eastern Europe & Central Asia	7.97	1.63	3.50	0.72	3.75	0.77	0.71	0.15
Kenya	Sub-Saharan Africa	0.50	0.22	0.35	0.15	0.13	0.06	0.03	0.01
Kiribati	Southeast Asia, East Asia, & Oceania	0.002	0.84	0.002	0.59	0.000	0.14	0.000	0.11
Kuwait	North Africa & Middle East	1.21	0.58	0.44	0.21	0.67	0.32	0.10	0.05
Kyrgyzstan	Central Europe, Eastern Europe & Central Asia	0.17	0.49	0.08	0.22	0.07	0.21	0.02	0.06
Laos	Southeast Asia, East Asia, & Oceania	0.43	0.76	0.29	0.51	0.11	0.19	0.03	0.06
Latvia	Central Europe, Eastern Europe & Central Asia	1.36	2.30	0.26	0.43	1.01	1.72	0.09	0.15
Lebanon	North Africa & Middle East	0.25	0.25	0.05	0.05	0.19	0.19	0.01	0.01
Lesotho	Sub-Saharan Africa	0.02	0.35	0.01	0.24	0.005	0.09	0.001	0.01

Liberia	Sub-Saharan Africa	0.009	0.13	0.006	0.09	0.002	0.03	0.000	0.006
Libya	North Africa & Middle East	0.61	0.59	0.18	0.18	0.39	0.38	0.04	0.03
Lithuania	Central Europe, Eastern Europe & Central Asia	1.97	1.91	0.38	0.37	1.42	1.38	0.17	0.17
Luxembourg	High Income	0.72	1.01	0.24	0.34	0.38	0.54	0.10	0.14
Madagascar	Sub-Saharan Africa	0.12	0.28	0.09	0.21	0.02	0.05	0.009	0.02
Malawi	Sub-Saharan Africa	0.02	0.11	0.01	0.07	0.007	0.03	0.001	0.007
Malaysia	Southeast Asia, East Asia, & Oceania	9.35	1.03	5.34	0.59	3.32	0.37	0.70	0.08
Maldives	Southeast Asia, East Asia, & Oceania	0.05	0.43	0.03	0.24	0.02	0.15	0.004	0.04
Mali	Sub-Saharan Africa	0.08	0.19	0.06	0.13	0.02	0.04	0.004	0.009
Malta	High Income	0.16	0.72	0.05	0.23	0.09	0.42	0.02	0.07
Mauritania	Sub-Saharan Africa	0.05	0.20	0.03	0.12	0.02	0.07	0.002	0.008
Mauritius	Southeast Asia, East Asia, & Oceania	0.32	1.11	0.15	0.52	0.13	0.44	0.04	0.14
Mexico	Latin America & Caribbean	9.08	0.36	3.69	0.15	3.62	0.14	1.77	0.07
Federated States of Micronesia	Southeast Asia, East Asia, & Oceania	0.003	0.66	0.002	0.47	0.000	0.11	0.000	0.07
Mongolia	Central Europe, Eastern Europe & Central Asia	0.64	1.62	0.53	1.34	0.07	0.16	0.05	0.12
Montenegro	Central Europe, Eastern Europe & Central Asia	0.33	2.50	0.26	1.90	0.07	0.53	0.009	0.07
Morocco	North Africa & Middle East	1.63	0.58	0.49	0.17	1.05	0.38	0.09	0.03
Mozambique	Sub-Saharan Africa	0.07	0.18	0.05	0.12	0.02	0.05	0.003	0.008
Myanmar	Southeast Asia, East Asia, & Oceania	2.51	0.91	1.64	0.60	0.70	0.26	0.17	0.06
Namibia	Sub-Saharan Africa	0.10	0.43	0.06	0.25	0.04	0.17	0.004	0.01
Nepal	South Asia	0.24	0.25	0.14	0.14	0.07	0.08	0.03	0.03
Netherlands	High Income	8.94	0.91	2.46	0.25	5.24	0.53	1.24	0.13
New Zealand	High Income	1.32	0.62	0.33	0.16	0.72	0.34	0.27	0.13
Nicaragua	Latin America & Caribbean	0.06	0.17	0.03	0.08	0.02	0.07	0.009	0.03
Niger	Sub-Saharan Africa	0.03	0.11	0.02	0.08	0.007	0.02	0.002	0.006
Nigeria	Sub-Saharan Africa	2.03	0.20	1.33	0.13	0.63	0.06	0.08	0.007
Republic of North Macedonia	Central Europe, Eastern Europe & Central Asia	0.89	2.56	0.26	0.77	0.54	1.57	0.08	0.22
Norway	High Income	3.02	0.88	0.68	0.20	1.81	0.53	0.52	0.15
Oman	North Africa & Middle East	0.56	0.41	0.21	0.15	0.30	0.22	0.05	0.04
Pakistan	South Asia	3.49	0.34	2.06	0.20	0.95	0.09	0.48	0.05
Panama	Latin America & Caribbean	0.71	0.53	0.31	0.23	0.27	0.20	0.14	0.10

Papua New Guinea	Southeast Asia, East Asia, & Oceania	0.20	0.51	0.14	0.36	0.03	0.07	0.03	0.07
Paraguay	Latin America & Caribbean	0.39	0.44	0.18	0.20	0.14	0.16	0.07	0.08
Peru	Latin America & Caribbean	1.17	0.28	0.51	0.12	0.39	0.09	0.27	0.06
Philippines	Southeast Asia, East Asia, & Oceania	6.64	0.69	4.41	0.46	1.70	0.18	0.53	0.06
Poland	Central Europe, Eastern Europe & Central Asia	17.11	1.36	4.80	0.38	10.44	0.83	1.87	0.15
Portugal	High Income	4.99	1.39	1.64	0.46	2.89	0.81	0.46	0.13
Puerto Rico	Latin America & Caribbean	0.75	0.68	0.30	0.27	0.35	0.32	0.10	0.09
Qatar	North Africa & Middle East	1.21	0.48	0.49	0.19	0.48	0.19	0.25	0.10
Moldova	Central Europe, Eastern Europe & Central Asia	0.58	1.66	0.23	0.68	0.31	0.90	0.03	0.09
Romania	Central Europe, Eastern Europe & Central Asia	15.13	2.62	4.74	0.82	8.97	1.55	1.42	0.25
Russia	Central Europe, Eastern Europe & Central Asia	91.81	2.30	23.98	0.60	59.68	1.49	8.15	0.20
Rwanda	Sub-Saharan Africa	0.05	0.19	0.04	0.13	0.01	0.04	0.003	0.01
Saint Lucia	Latin America & Caribbean	0.02	0.84	0.01	0.41	0.010	0.35	0.002	0.09
Saint Vincent and the Grenadines	Latin America & Caribbean	0.01	0.81	0.006	0.42	0.004	0.32	0.001	0.06
Samoa	Southeast Asia, East Asia, & Oceania	0.010	0.78	0.007	0.51	0.002	0.18	0.001	0.08
São Tomé and Príncipe	Sub-Saharan Africa	0.003	0.32	0.002	0.21	0.001	0.10	0.000	0.01
Saudi Arabia	North Africa & Middle East	19.29	1.20	9.48	0.59	8.89	0.55	0.92	0.06
Senegal	Sub-Saharan Africa	0.11	0.20	0.08	0.14	0.03	0.06	0.005	0.009
Serbia	Central Europe, Eastern Europe & Central Asia	3.44	2.71	0.73	0.57	2.44	1.92	0.28	0.22
Seychelles	Southeast Asia, East Asia, & Oceania	0.03	1.20	0.02	0.67	0.01	0.46	0.002	0.07
Sierra Leone	Sub-Saharan Africa	0.03	0.19	0.02	0.13	0.007	0.05	0.001	0.008
Singapore	High Income	4.97	0.89	1.84	0.33	2.28	0.41	0.85	0.15
Slovakia	Central Europe, Eastern Europe & Central Asia	2.27	1.30	0.60	0.35	1.48	0.85	0.19	0.11
Slovenia	Central Europe, Eastern Europe & Central Asia	0.84	1.04	0.22	0.27	0.53	0.65	0.09	0.11
Solomon Islands	Southeast Asia, East Asia, & Oceania	0.01	0.83	0.01	0.64	0.002	0.11	0.001	0.08
South Africa	Sub-Saharan Africa	3.25	0.44	1.65	0.23	1.48	0.20	0.12	0.02
Spain	High Income	14.76	0.77	5.08	0.26	7.76	0.40	1.91	0.10
Sri Lanka	Southeast Asia, East Asia, & Oceania	1.72	0.60	0.61	0.22	0.89	0.31	0.21	0.08

Sudan	North Africa & Middle East	0.51	0.28	0.19	0.11	0.28	0.16	0.03	0.02
Suriname	Latin America & Caribbean	0.11	1.06	0.06	0.63	0.03	0.33	0.010	0.10
Sweden	High Income	5.09	0.94	1.28	0.24	3.25	0.60	0.56	0.10
Switzerland	High Income	4.27	0.73	0.98	0.17	2.58	0.44	0.71	0.12
Tajikistan	Central Europe, Eastern Europe & Central Asia	0.10	0.33	0.08	0.26	0.02	0.05	0.004	0.01
Thailand	Southeast Asia, East Asia, & Oceania	12.86	1.00	6.71	0.52	4.13	0.32	2.02	0.16
Timor-Leste	Southeast Asia, East Asia, & Oceania	0.02	0.46	0.01	0.29	0.006	0.14	0.001	0.03
Togo	Sub-Saharan Africa	0.02	0.17	0.02	0.12	0.005	0.04	0.001	0.007
Tonga	Southeast Asia, East Asia, & Oceania	0.003	0.38	0.001	0.21	0.001	0.13	0.000	0.04
Trinidad and Tobago	Latin America & Caribbean	0.36	0.98	0.14	0.39	0.17	0.47	0.05	0.12
Tunisia	North Africa & Middle East	0.71	0.57	0.18	0.14	0.49	0.39	0.04	0.03
Turkey	North Africa & Middle East	17.26	0.73	6.48	0.28	8.86	0.38	1.93	0.08
Turkmenistan	Central Europe, Eastern Europe & Central Asia	1.01	1.10	0.50	0.55	0.40	0.43	0.11	0.12
Uganda	Sub-Saharan Africa	0.12	0.12	0.08	0.08	0.03	0.03	0.007	0.008
Ukraine	Central Europe, Eastern Europe & Central Asia	9.35	1.74	2.74	0.51	5.89	1.09	0.73	0.13
United Arab Emirates	North Africa & Middle East	8.69	1.32	3.35	0.51	4.74	0.72	0.60	0.09
United Kingdom	High Income	25.55	0.82	7.23	0.23	13.87	0.45	4.45	0.14
United States	High Income	214.61	1.04	70.55	0.34	106.67	0.52	37.39	0.18
Uruguay	High Income	0.64	0.87	0.18	0.25	0.34	0.46	0.12	0.16
Uzbekistan	Central Europe, Eastern Europe & Central Asia	1.51	0.64	0.83	0.35	0.60	0.25	0.08	0.03
Vanuatu	Southeast Asia, East Asia, & Oceania	0.006	0.67	0.004	0.46	0.001	0.14	0.001	0.07
Vietnam	Southeast Asia, East Asia, & Oceania	8.43	1.09	5.09	0.66	2.84	0.37	0.50	0.06
Zambia	Sub-Saharan Africa	0.17	0.27	0.12	0.19	0.04	0.06	0.009	0.01
Zimbabwe	Sub-Saharan Africa	0.07	0.18	0.03	0.08	0.03	0.07	0.009	0.02

VLW = Value of Lost Welfare, GDP = Gross Domestic Product.
